# Functional Characterization of Invertase Inhibitors PtC/VIF1 and 2 Revealed Their Involvements in the Defense Response to Fungal Pathogen in *Populus trichocarpa*

**DOI:** 10.3389/fpls.2019.01654

**Published:** 2020-01-08

**Authors:** Tao Su, Mei Han, Jie Min, Huaiye Zhou, Qi Zhang, Jingyi Zhao, Yanming Fang

**Affiliations:** ^1^ Co-Innovation Center for Sustainable Forestry in Southern China, College of Biology and the Environment, Nanjing Forestry University, Nanjing, China; ^2^ Key Laboratory of State Forestry Administration on Subtropical Forest Biodiversity Conservation, Nanjing Forestry University, Nanjing, China; ^3^ College of Forest, Nanjing Forestry University, Nanjing, China

**Keywords:** poplar, invertase inhibitor, sucrose, apoplast, pathogen, defense response, drought

## Abstract

In higher plants, cell wall invertase (CWI) and vacuolar invertase (VI) were considered to be essential coordinators in carbohydrate partitioning, sink strength determination, and stress responses. An increasing body of evidence revealed that the tight regulation of CWI and VI substantially depends on the post-translational mechanisms, which were mediated by small proteinaceous inhibitors (C/VIFs, Inhibitor of β-Fructosidases). As yet, the extensive survey of the molecular basis and biochemical property of C/VIFs remains largely unknown in black cottonwood (*Populus trichocarpa* Torr. & A. Gray), a model species of woody plants. In the present work, we have initiated a systematic review of the genomic structures, phylogenies, *cis*-regulatory elements, and conserved motifs as well as the tissue-specific expression, resulting in the identification of 39 genes encoding C/VIF in poplar genome. We characterized two putative invertase inhibitors *PtC/VIF1* and *2*, showing predominant transcript levels in the roots and highly divergent responses to the selected stress cues including fusarium wilt, drought, ABA, wound, and senescence. *In silico* prediction of the signal peptide hinted us that they both likely had the apoplastic targets. Based on the experimental visualization *via* the transient and stable transformation assays, we confirmed that PtC/VIF1 and 2 indeed secreted to the extracellular compartments. Further validation of their recombinant enzymes revealed that they displayed the potent inhibitory affinities on the extracted CWI, supporting the patterns that act as the typical apoplastic invertase inhibitors. To our knowledge, it is the first report on molecular characterization of the functional C/VIF proteins in poplar. Our results indicate that PtC/VIF1 and 2 may exert essential roles in defense- and stress-related responses. Moreover, novel findings of the up- and downregulated C/VIF genes and functional enzyme activities enable us to further unravel the molecular mechanisms in the promotion of woody plant performance and adapted-biotic stress, underlying the homeostatic control of sugar in the apoplast.

## Introduction

Sucrose synthesized in source leaves represents the primary form of carbon assimilates translocated *via* the phloem complex to non-photosynthetic sink organs (Koch, 2004). During the passage, two classes of sucrose-splitting enzymes intermediate the sucrose hydrolysis. Sucrose synthase (EC2.4.1.13, Susy) reversibly converts sucrose into UDP-glucose and fructose, both of which are utilized for the cell respiration and cellulose biosynthesis (Coleman et al., 2009). By contrast, invertase (EC 3.2.1.26) irreversibly catalyzes the cleavage of sucrose into its hexose (glucose and fructose) components, exerting a pivotal role in carbon utilization and distribution. After unloading into sink cells, sucrose is either taken up symplastically by intracellular trafficking pathway *via* plasmodesmata for the metabolic and synthetic processes (Rausch and Greiner, 2004), or it can also be apoplastically transported by sucrose transporters (SUTs) to the extracellular space for fungal colonization and defense-related responses (Roitsch et al., 2003; Doidy et al., 2012).

Evolutionary analyses between various cellular organisms suggested the presence of two smaller sub-families, acid invertase (AI) and cytosolic neutral/alkaline invertase (CI) distinguished by the properties of protein solubility, pH optima, and subcellular targets (Sturm, 2002; Wan et al., 2018). The AI sub-family is comprised of cell wall invertase (CWI) and vacuolar invertase (VI). The deduction of protein structure and domain revealed that CWI and VI are clustered to GH32 (glycoside hydrolase family 32) enzymes with an optimal pH of 3.5–5.0, sharing similar patterns of conserved motifs and catalytic domains (Van den Ende et al., 2009). It is worthwhile to note that AIs are all glycosylated enzymes and intrinsically stable; however, CI varies substantially from AI in molecular and biochemical properties and belongs to GH100 with an optimal pH of 6.8–9.0, appearing to be localized to cytosols, mitochondrion, plastids, and nucleus.

It has been long known that CIs compensate for the loss of Susy and AI activities, fulfilling roles in sucrose metabolism (Liu et al., 2015), cellulose biosynthesis (Rende et al., 2017; Barnes and Anderson, 2018), nitrogen uptake (Tamoi et al., 2010; Maruta et al., 2015), and reactive oxygen species (ROS) scavenging as well as osmotic stress adaptation (Xiang et al., 2011; Battaglia et al., 2017). However, AIs playing multi-faceted actions in source–sink interactions have received much more attention. The hexoses released by CWI or VI not only served as core metabolites and nutrient sources but also acted as key signaling molecules to impact on gene expression during developmental transitions and responding to environmental cues (Rolland et al., 2006; Ruan, 2014). The basic functions of VI in photoassimilate partitioning, cell expansion, and osmotic regulation have been implemented widely in a variety of plants (Klann et al., 1996; Kohorn et al., 2006; Sergeeva et al., 2006; Yu et al., 2008; Nägele et al., 2010; Morey et al., 2018). Suppression of VI activities showed a decrease of cold-induced sweetening (CIS), leading to improved processing qualities of potato tubers (Bhaskar et al., 2010; Zhu et al., 2016). Aside from the developmental functions, VI exerts important roles in stress tolerance (e.g. drought and cold) by sustaining the homeostasis of sugar metabolism (Qian et al., 2018; Weiszmann et al., 2018; Wei et al., 2019).

By contrast, apoplastic CWI splits sucrose into hexose components that were further translocated either into intracellular compartments for the transcriptional regulation, sugar metabolism, and polysaccharide biosynthesis or into extracellular space for the enhancement of sink capacity and stress responses (Bihmidine et al., 2013; Proels and Hückelhoven, 2014). The promotions of CWI on seed filling and fruit set have been well attempted in a wide range of plant species like maize, rice, tomato, cotton, and litchi (Chourey et al., 2006; Wang et al., 2008; Zanor et al., 2009; Wang and Ruan, 2012; Li et al., 2013; Zhang et al., 2018), indicating that CWIs facilitate the improvement of sink cell differentiation *via* multiple regulatory mechanisms of sugar metabolism and signaling. Recently, overexpressing *CWIs* in tobacco and tomato resulted in the deferral of leaf aging and drought avoidance (Balibrea Lara et al., 2004; Albacete et al., 2015). Also numerous reports also revealed that CWI plays central roles in defense and immune responses during plants–pathogen interactions (Swarbrick et al., 2006; Essmann et al., 2008; Kocal et al., 2008; Sun et al., 2014; Veillet et al., 2016), pointing out that CWI serves as a significant stress indicator and pathogenesis-related proteins.

Early research focused primarily on the induction of AI activities through the (post-) transcriptional increases in their corresponding gene transcripts (Ehness and Roitsch, 1997). However, given the protein glycosylation and discordant protein/transcript expression patterns, the tight control of AI may subject primarily to the post-translational mechanisms. Accumulating evidence has confirmed that CWI and VI activities were explicitly determined by the low-molecular-weight (15–23 kDa) proteinaceous inhibitors, namely C/VIFs (cell wall/vacuolar inhibitor of β-fructosidases) according to the targeting patterns. *In silico* analyses revealed that the C/VIF family is moderately conserved within one species and various plant species (Rausch and Greiner, 2004). C/VIFs and the structure-related PMEIs (pectin methylesterase inhibitors) belong to the same superfamily, enabling it with difficulties to distinguish them from sequence comparisons (Hothorn et al., 2004). However, an enigma of whether C/VIFs have genuine *in vivo* inhibitory activities against the targeted enzymes remains to be unlocked. Using the heterologous expression system, some CIFs were functionally dug out in tobacco, tomato, and maize (Greiner, 1998; Bate, 2004; Reca et al., 2008). After that, crystal analyses of complex uncovered that CIF used its small motifs (PKF) to target CWI through physical binding to substrate cleft in a pH-dependent manner (Hothorn et al., 2004; Hothorn et al., 2010).

Overexpression of VIF-encoded genes led to the insensitivity to CIS of potato tubers (Greiner et al., 1999; Brummell et al., 2011; Liu et al., 2013; Mckenzie et al., 2013), indicating that the functional capping VI restrained glucose release *via* the post-translational regulation. In addition to biotechnology relevance, recent reports suggested that the VIF-mediated sucrose metabolism conferred the alterations of fruit ripeness and drought stress tolerance (Chen et al., 2016; Qin et al., 2016). By contrast, silencing of *CIF* expression also facilitated the improvements of seed filling, prolonged leaf green, and cold tolerance in tomato (Jin et al., 2009; Xu et al., 2017), highlighting that the post-translational control is necessary for hexoses to release to sink organs, particularly responding to stressors and phytohormone cues. These results corroborated the findings that the suppression of *CIF* expression resulted in increased seed production and germination (Su et al., 2016; Tang et al., 2017). An increasing body of evidence supported the notion that the C/VIF-mediated post-translational modulation of invertase commonly involves multiple cellular processes, metabolic pathways, and molecular regulation. Interestingly, the post-translational elevation of CWI activities and its components by native inhibitors in *Arabidopsis* contributed to a marked reduction of susceptibility and disease index to the bacterial and fungal pathogens (Bonfig et al., 2010; Siemens et al., 2011), indicating that the rapid rise of CWI acted as a significant signal of defense during the plant host and pathogen interactions.

Poplar has served as a model woody organism in perennial plants and forestry for research of biology and molecular physiology owning to high superiorities for plantation, biomass, and ecological functions (Jansson and Douglas, 2007). Despite the advances that have been made in a variety of plant species, little was known about genes encoding C/VIF and the enzyme properties in *P. trichocarpa*. In a bid to rectify this situation, we conducted a genome-wide survey of C/VIF candidates in the recently released genome of *P. trichocarpa* (Tuskan et al., 2006). Based on the conserved patterns and expression profiling, we reported the molecular isolation and functional characterization of two PtC/VIFs using the bacterial expressed recombinant proteins. Their subcellular targets were explored by ectopic expression of fluorescent fusions *via* transient and stable assays. Here, the demonstrated substantial regulation of gene transcripts upon various stressors concurrent with enzyme targeting activities provided a promising strategy for the future unraveling the *in vivo* roles of C/VIF family in poplar and other woody perennials.

## Materials and Methods

### Plant Materials, Growth Conditions, and Stress Treatments

*P*. *trichocarpa* (genotype Nisqually-1) grows on standard pot in the growth chamber, using a temperature cycling between 22°C (night) and 26°C (day) under long-day conditions (16 h light/8 h dark, 20 μE) according to a previous report (Li et al., 2017; Su et al., 2019). *N. benthamiana* and *A. thaliana* (ecotype *Col-0*) plants were maintained in a growth chamber at 25°C under a light regime of 16 h and 200–300 μE of long-day conditions. Unless otherwise specified, vegetative tissues of eight-week-cultured *P*. *trichocarpa* and floral organs of field-grown *P. deltoids* were harvested for qRT-PCR according to a previous study (Bocock et al., 2008). The *in vitro P*. *trichocarpa* were cultured (25°C, 16/8 h day/night photoperiod, 20 μE) on wood plant medium (WPM) with 30 g l^−1^ sucrose, 0.1 mg l^−1^ IBA, and solidified with 8 g l^−1^ plant agar (Biofroxx). After a culturing for five weeks, seedlings were transferred to standard pots with mixtures of vermiculite:perlite:peat (1:1:3). For the infection of the fungal pathogen, roots peripheral areas of 8-week-cultured plants were inoculated with 20 ml *F. solani* spore suspensions (2.0 × 10^6^ spore/ml) for 48 and 72 h. Similarly, plants were irrigated with 20 ml ABA (100 μM, dissolved in 10% ethanol) once a day for 4 days, and grown for 48 and 96 h. The drought stress was induced by water withholding treatments for 96 and 120 h. For wounding treatments, the mature leaves were physically punched and harvested after 2 and 6 h. Seasonal senescence leaves were harvested from plants grown in a growth chamber according to a previous study (Su et al., 2019). The frozen samples were ground in the liquid nitrogen and subjected to RNA and protein extraction, followed by qRT-PCR and functional assay.

### Sequence Available, Gene Structure and Distribution, *Cis*-Element, and Conserved Motif

The previously described C/VIFs (Link et al., 2004; Tang et al., 2017) were collected as queries to search for the homologs in *P. trichocarpa* genome assembly (3.0) from the JGI gene catalog (Phytozome v12.1, https://phytozome.jgi.doe.gov/pz/portal.html) with the *E*-value cutoff set as 1e-5 and GenBank (https://www.ncbi.nlm.nih.gov/). The respective protein sequences were verified in Pfam (http://pfam.xfam.org/) by the HMMER program (3.1b2). The incomplete sequences with too short (<150 aa) and too long (>250 aa) length as well as sequences showing more than 98% identities were eliminated. The genomic structure was deduced by comparing the coding sequences (CDS) and corresponding DNA sequences using the GSDS (Hu et al., 2015). The chromosomal distribution of *PtC/VIFs* candidates was obtained from the PopGenIE (http://popgenie.org/chromosome-diagram) and was drawn with MapInspect (http://www.softsea.com/review/MapInspect.html). The conserved motifs were identified by the MEME program (http://meme-suite.org/index.html) with default settings except that the maximum widths of motifs were set to 50 (Bailey et al., 2006). Approximately 1.5-kb upstream regions were used to search for the *cis*-acting regulatory elements in the PlantCARE (http://bioinformatics.psb.ugent.be/webtools/plantcare/html/). The putative transcription factor (TF) binding sites were analyzed in the PlantTFDB 4.0 (http://planttfdb.cbi.pku.edu.cn/). Signal peptides and subcellular targeting sequences were deduced by online programs of PSORT (https://wolfpsort.hgc.jp/) and Phobius (http://phobius.binf.ku.dk/).

### Transcriptomic Sequencing and Expression Analysis

Transcriptomic sequencing (RNA-seq) of eighteen vegetative and reproductive tissues (SRA: SRP077540) was collected from Phytozome (v12.1) (BioProject: PRJNA10772; Accession number: GCF_000002775.4). The Affymetrix expression data (BioProject: PRJNA112485; GEO: GSE13990) is accessible from Poplar eFP Browser (http://bar.utoronto.ca/efppop/cgi-bin/efpWeb.cgi). Duplicate or triplicate samples of *P. trichocarpa* were used for microarray analysis (Wilkins et al., 2009). For the qRT-PCR analyses, RNA extraction and cDNA synthesis were performed according to the previous report (Han et al., 2013; Su et al., 2019). Total RNA was extracted using the RNeasy Plant Mini Kit (Qiagen, China). RNase-free DNase I (Qiagen, China) was used to remove genomic DNA. First-strand cDNA was synthesized using the PrimeScript II 1st Strand cDNA Synthesis Kit (Takara, China). For a standard qRT-PCR assay, samples were loaded to a TB green Premix ExTap™ Tli RNaseH Plus (Takara, China). The mixture was subjected to StepOnePlus™ Real-Time PCR System (AB, USA) with a three-step PCR using the cycling parameters: 95°C for 30 s, followed by 95°C for 5 s and 60°C for 30 s, for 40 cycles, and a melt cycle from 65 to 95°C. The primer amplification efficiency was evaluated with dilutions of cDNA, producing an R^2^ value ≥ 0.99. The relative expression of the target gene was normalized by the geometric mean (Vandesompele et al., 2002) of three reference genes: *PtUBIC*, *Ptβ-Actin*, and *PtEF-1α*. The detailed primers used for targeting specific genes are listed in **Supplementary Table S1**.

### Plant Transformation

The *Agrobacterium*-mediated transformation in *Arabidopsis* by floral dip has been described previously (Clough and Bent, 1998). Transformants were primarily screened by spraying BASTA^®^ on seedlings grown in soil. The T2 homozygous generations were used for image analysis as indicated. For the transient transformation in tobacco (*N. benthamiana*), the *Agrobacterium* strain (*C58C1*) containing the appropriate constructs were grown overnight in 30 ml of YEB-medium supplemented with carbenicillin (50 μg ml^–1^), rifampicin (100 μg ml^–1^) and spectinomycin (50 μg ml^–1^) until the stationary phase. After centrifugation at 3000 g for 30 min, the cells were re-suspended in 15 ml of infiltration buffer [10-mM 2-(N-morpholino) ethanesulfonic acid (MES), pH 5.9, 150-μM acetosyringone] and incubated with gentle agitation for 2 h at room temperature. The suspension cells were mixed with infiltration buffer and adjusted to OD600 = 1.0. The lower epidermis of 5-week-old tobacco leaves was infiltrated with *Agrobacterium* via a needless syringe. After two days of inoculation, the transformed regions were subject to confocal laser scanning microscopy (CLSM) for image analyses.

### Subcellular Localizations

The analyses of subcellular localization were conducted according to a previous study (Tang et al., 2017). The CDS (stop codon omitted) of *PtC/VIF1* and *PtC/VIF2* were amplified by PCR using the primers containing the Gateway™ (Invitrogen, Germany) *attB1* and *attB2* recombinant sites (**Supplementary Table S1**). The respective PCR products were recovered and then inserted into the donor plasmid *pDONR201* and subsequently recombined with the binary destination vector *pB7YWG2.0*, yielding the *pB7C/VIF1-YFP* and *pB7C/VIF2-YFP* constructs. Half of the tobacco leaves were co-infiltrated with *A. tumefaciens* (*C58C1*), harboring the C-terminal YFP-fusion constructs and the *Arabidopsis* cell wall-localization marker (*pK7CIF1-RFP*). As a control, another half leaves were infiltrated with strain with null constructs. The visualization of fluorescent signals in the transgenic *Arabidopsis* roots was conducted according to a previous study (Su et al., 2016). The YFP was excited by a 514 nm laser line and the emitted fluorescent signal was collected by a 530–600 nm bandpass filter. The RFP was excited with a 543 nm laser line, and the emitted fluorescence was captured with a 560 nm long-pass filter. Images were analyzed by a Zeiss LSM 510 Meta inverted CLSM.

### Heterologous Expression and Purification of PtC/VIF1 and 2

The protein purification was performed according to the previous reports (Link et al., 2004; Tang et al., 2017). The CDS (signal peptide omitted) of *PtC/VIF1* and *2* were amplified using primers containing the Gateway™ (Invitrogen, Germany) *attB1* and *attB2* recombinant sites (**Supplementary Table S1**) from the roots, followed by recombination with the destination vector *pETG-20A*, yielding 6x His-tagged thioredoxin A (TrxA) fusion constructs that were introduced into the *E. coli* strain Rosetta-gami™ (DE3) (Novagen, Germany) for recombinant protein induction and expression. Bacterial cells were harvested by centrifugation at 10,000 g for 15 min and lysed with 1/20 volume of lysis buffer (50-mM Na_2_HPO_4_/NaH_2_PO_4_, pH 7.0, 500-mM NaCl, 1% Triton X-100, 1 mg ml^–1^ lysozyme) and repeat the centrifugation at 15 000 g for 1 h. The supernatant was collected and mixed with 0.6 g Ni-TED Protino resin (Macherey-Nagel, Germany) and kept stirring at 4°C for 45 min to enable protein binding. After loading to the column, the resin was firstly washed with lysis buffer followed by washing buffer (50-mM Na_2_HPO_4_/NaH_2_PO_4_, pH 7.0, 500-mM NaCl, 10% glycerol). The bound TrxA-fusion proteins were then eluted with 10 volumes of the imidazole (250 mM) containing a washing buffer. Afterward, the eluted proteins were dialyzed against TEV protease cleavage buffer (50-mM Na_2_HPO^4^/NaH_2_PO_4_, pH 7.0, 200-mM NaCl) at 30°C for 3 h before loading to the column. A second elution was conducted to eliminate 6× His tags, yielding the finally purified recombinant proteins.

### Invertase Extraction and Functional Assay

The acid invertase (CWI and VI) extraction and the functional assay were conducted according to the previous reports (Link et al., 2004; Tang et al., 2017). For CWI preparation, the root tissues were ground in the liquid nitrogen and homogenized in 500-μl extraction buffer (30-mM MOPS, 250-mM sorbitol, 10-mM MgCl_2_, 10-mM KCl, and 1-mM PMSF, pH 6.0). After centrifugation for 10 min (8 000g, 4°C), the insoluble cell wall pellets were washed once with extraction buffer plus 1% Triton X-100, and twice with extraction buffer only, followed by the re-suspension in 500-μl assay buffer (20-mM triethanolamine, 7-mM citric acid, and 1-mM PMSF, pH 4.6). For VI preparation, endogenous sucrose in the soluble fraction was removed by precipitation of 4 volumes of ice-cold acetone (–20°C, 20 min). After centrifugation for 10 min (15,000 g, 4°C), the pellets were resolved in 1 volume of assay buffer. The inhibitory activities of recombinant proteins were determined against the extracted CWI and VI. Variable amounts of purified recombinant proteins were mixed with suitable invertase preparations in assay buffer. A total amount of 200 μl mixtures was incubated at 37°C for 30 min to enable the complex formation and then mixed with 100 μl sucrose (100 mM) for 60 min. The reactions were terminated by sodium phosphate buffer (1 M, pH 7.5) and quickly boiled at 95°C for 5 min. The liberated glucose was quantitated by a coupled enzymatic-optical assay according to the Lambert–Beer Law and the enzyme activity was expressed in nkat g^–1^ fresh weight (1 nkat = 1 nmole glucose liberated/second). Each experiment was performed in a quadruplicate, one of which without the addition of the recombinant protein was calculated as the background of absorption.

## Results

### Genome-Wide Identification of the Invertase Inhibitor Genes in *P. trichocarpa*

**Figure 1:**
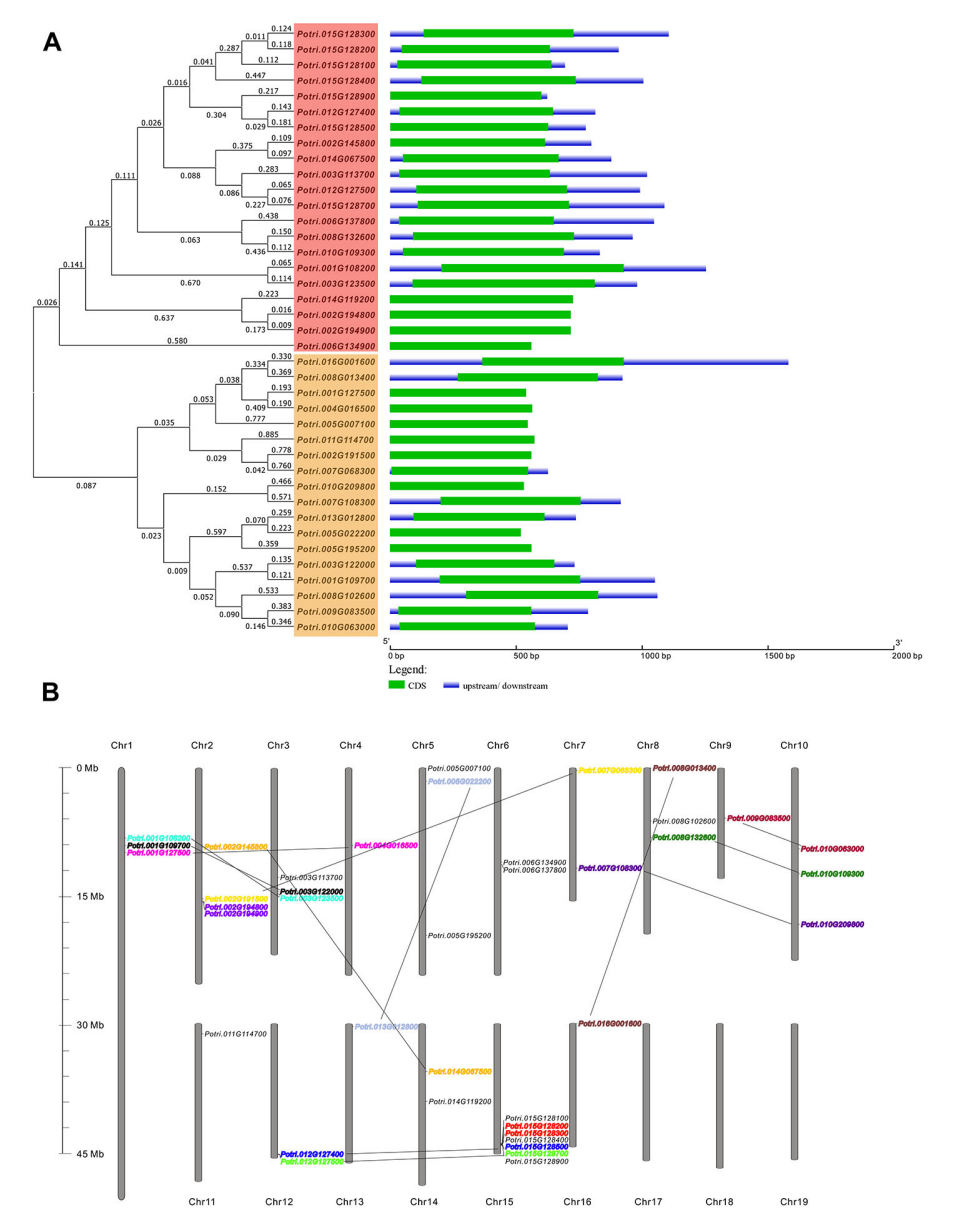
Genomic structures and the chromosomal distribution of *PtC/VIF* candidate genes. **(A)** Gene structures showing the exon/intron organization that was analyzed by the online tool GSDS. The full-length sequences of mRNA were aligned by ClustalW Omega (https://www.ebi.ac.uk/Tools/msa/clustalo/) to generate the Neighbour-joining tree with branched length by a cladogram, and on the left, the gene classification was indicated. The lengths of exons are displayed proportionally to the scale on the bottom **(B)** Thirty-nine *PtC/VIF/PMEIs* were anchored on 16 chromosomes. Pairs of gene speculated to have undergone segmental/tandem duplication are lined and labeled in the same color.

Using the reported C/VIFs in *Arabidopsis* and soybean as queries, the systematic BLAST was performed in Phytozome (v.12.1) database, retrieving a large number of homologs within the genome (v3.0) of *P. trichocarpa*. After removal of the redundant sequences, a total of 39 genes encoding C/VIF were identified and postulated to be as *C/VIF* candidates. As we are not able to distinguish C/VIF from PMEI based on the conserved sequence alone, all members were annotated as C/VIF/PMEI superfamily genes in our analyses. The accession ID, chromosomal location, CDS and open reading frame, protein size, molecular weights (MWs), isoelectric point (pI), deduced signal peptide, and subcellular targets are analyzed. We found that all members had no presence of the transcript variants (**Supplementary Table S2**). The translated protein sequences varied from 172 to 241 amino acid residues with theoretical MWs ranging from 18.42 to 26.60 kDa. Most of C/VIF candidates were predicted to contain the targeting peptides. An unrooted phylogenetic tree revealed that the *C/VIF* candidates were divided into two sub-families (**Figure 1A**). Further comparison of genomic structure and exon/intron organization revealed that they are all encoded by only one exon, whose length and locations are generally conserved (**Figure 1A**). Patterns of chromosomal locations revealed that all members were mapped on sixteen out of the 19 chromosomes (Chr) with the individual distribution from Chr1 to Chr16 (**Figure 1B**). Additionally, twice genome duplication events were assumed to occur in poplar (Tuskan et al., 2006). Based on the phylogenetic analyses, the 14 pairs of genes were clustered together with high protein sequence identities, of which two pairs of genes (Potri.015G128200/300 and Potri.002G194800/900) were identified to likely evolve as the consequence of tandem duplication as they were adjacent on a chromosome segment.

**Figure 2:**
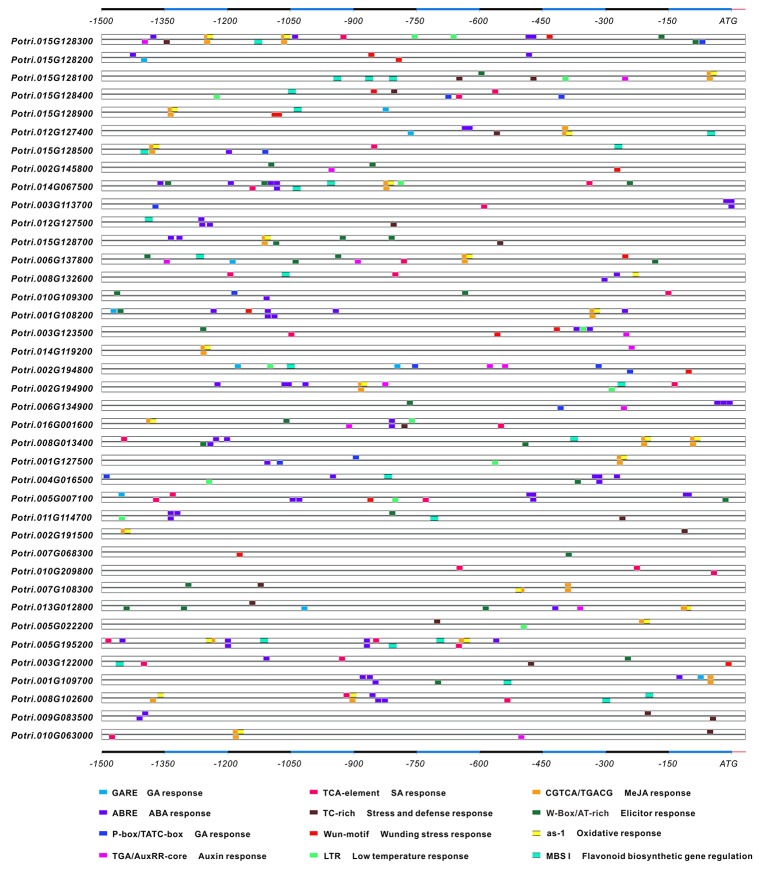
The *in silico* prediction of the *cis*-regulatory elements in the promoters. Upstream 1.5 kb sequences of each gene promoter were analyzed in the PlantCARE server. The stress- and phytohormone-related *cis*-regulatory elements are boxed in different colors.

Regulatory elements within the gene promotor are the essential clues to characterize the environmental stimuli that modulate gene expression. *In silico* prediction was conducted in the PlantCARE database, resulting in findings of seven *cis*-regulatory elements associated with phytohormone regulation, and five of which involved in stress- and defense-related responses (**Figure 2**). ABA-responsive elements (ABRE) were found to more widely spread in the promotors of 27 genes, followed by the jasmonate (MeJA)-responsiveness elements (TGACG and CGTCA) and salicylic acid (SA)-responsive elements (TCA), which were identified in 20 genes. However, the gibberellin-responsive elements (GARE-motif, P-box, and TATC-box) and auxin-responsive elements (AuxRR-core/TGA-element) were rich in a small number of genes. By contrast, a few defense and stress-related *cis*-acting elements, including wounding (WUN-motif), TC-rich repeats, low temperature (LTR), and oxidation (as-1) were abundantly distributed in 10 to 16 genes. The TFs of MYB binding sites involved in carbon metabolism were listed. The characterization of prevalent *cis*-regulatory elements and the TF binding sites provided the clue that the molecular regulation of genes may depend on the crosstalk between phytohormones, stress, and nutrient sources.

### Mining Conserved Motifs, Phylogenetic Evolution, and Expression Profiling

**Figure 3:**
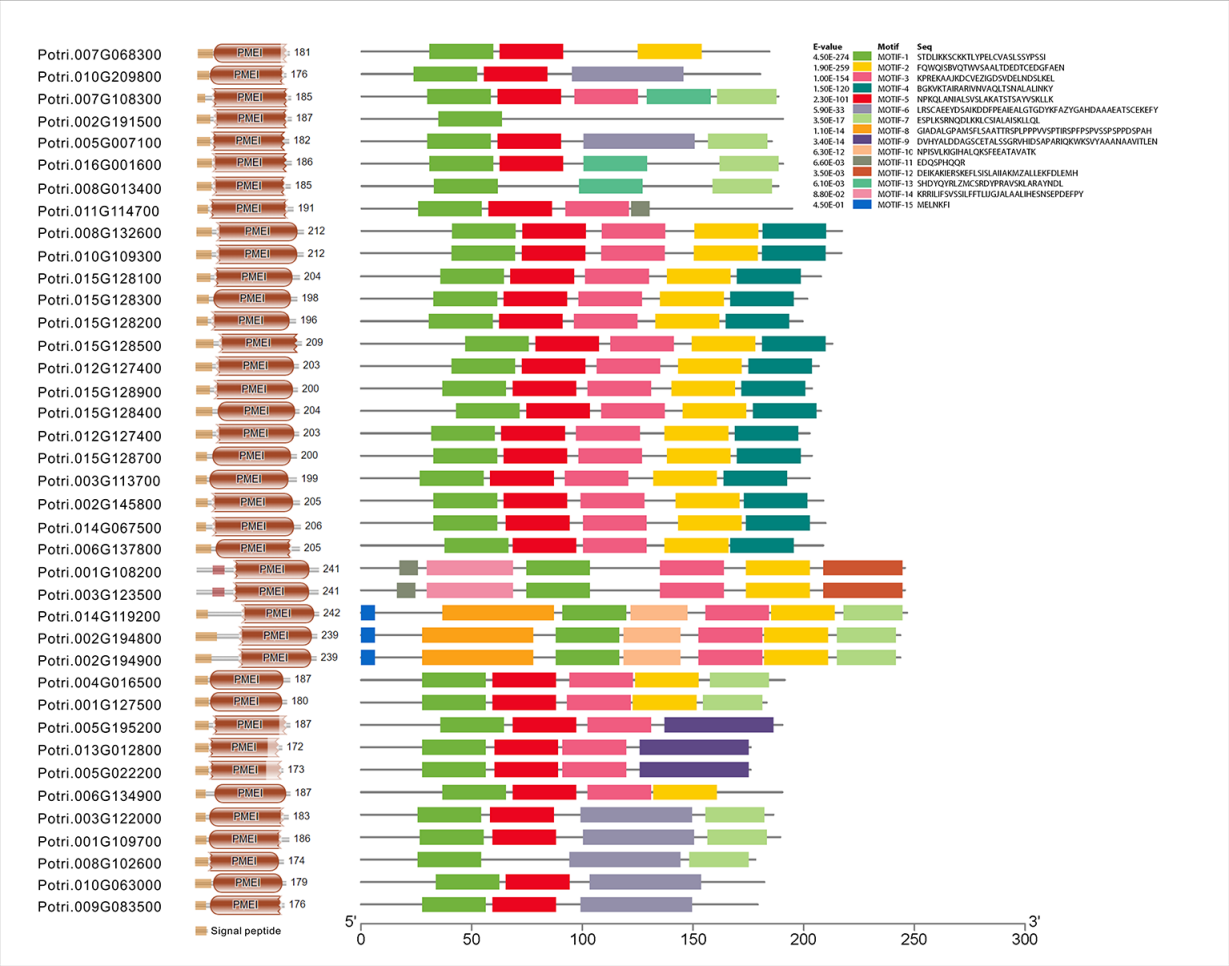
The deduction of conserved motifs and amino acid residues. The distribution of signal peptides, conserved domain, and 15 motifs of 39 PtC/VIF/PMEIs was programmed by Pfam (32.0) and MEME. The motif-1 contains the first pair of Cys residues that were characterized to be involved in the formation of the disulfide bridge.

**Figure 4:**
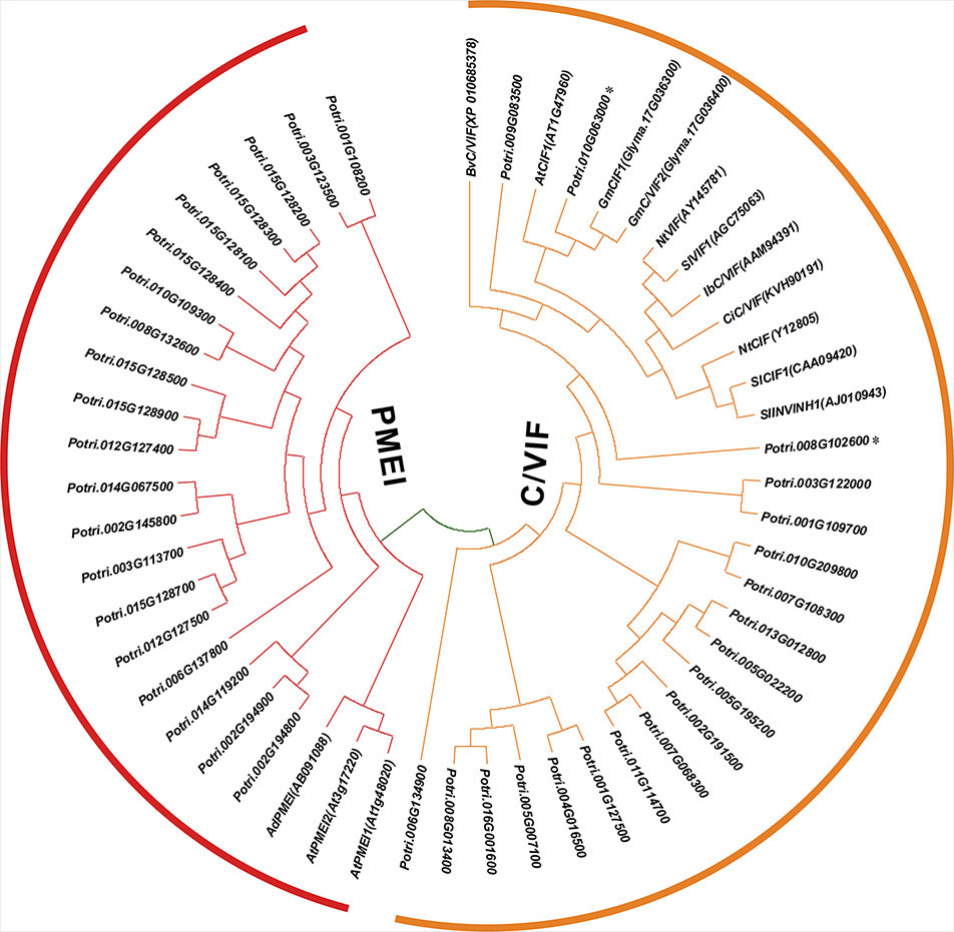
Phylogenetic relationships of PMEI and C/VIF homologs between poplar and other plant species. Multiple protein sequences of PMEI/C/VIF were aligned with the other nine plant species by ClustalW. The unrooted phylogenetic tree was constructed by MEGA7 (https://www.megasoftware.net/) using the neighbor-joining method (Kumar et al., 2016). The evolutionary distances were computed using the Poisson correction method and are in the units of the number of amino acid substitutions per site. The percentage of replicate trees in which the associated taxa clustered together in the 1000 bootstrap test is shown next to the branches. The experimentally verified *PMEI* and *C/VIF* were reported in *N. tabacum* (Nt), *A. thaliana* (At), *B. vulgaris* (Bv), *I. batatas* (Ib), *C. intybus* (Ci), *G. max* (Gm), *S*. *lycopersicum* (Sl), *S. tuberosum* (St), and *A. deliciosa* (Ad). The accession numbers in Genbank are adjacent to the corresponding genes.

To gain insight into the conserved patterns, the full-length protein sequences were analyzed by Pfam (32.0) and MEME. Both C/VIF and PMEI family are homologous inhibitors containing targeting sequences and four cysteines (Cys) residues that have been verified the formation of two strictly conserved disulfide bridges to strengthen protein structure. All C/VIF candidates showed the same conserved PMEI/C/VIF domain (IPR035513; IPR034087), which is annotated with the functional inhibition on PME and/or invertase activities (**Figure 3**). Accordingly, a total of 15 sequence fragments were programmed to be as the putatively conserved motifs by MEME analyses. Interestingly, the motif-1 deduced in all members contains the first pair of Cys residues with the random insertion of eight amino acids. Other motifs containing the third and fourth Cys residues varied in the presence from 24 to 28 members (**Figure 3**). To further assess the evolutionary relationship and distinct origin, all members were aligned with the reported C/VIF and PMEI genes in the other nine plant species. The alignment of the full-length protein sequences revealed that all 53 homologs were categorized into two distinct sub-clades with well-supported bootstrap values, termed PMEI family and C/VIF family (**Figure 4**). Five of 19 *PtC/VIF* candidates within the C/VIF sub-clades were identified to be evolutionarily close to the three confirmed C/VIF paralogs in *Arabidopsis*, soybean, and sugar beet. By contrast, three of 20 *PtPMEI* candidates displayed similarities with genes in *Arabidopsis* and kiwi within the PMEI sub-clade (**Figure 4**).

**Figure 5:**
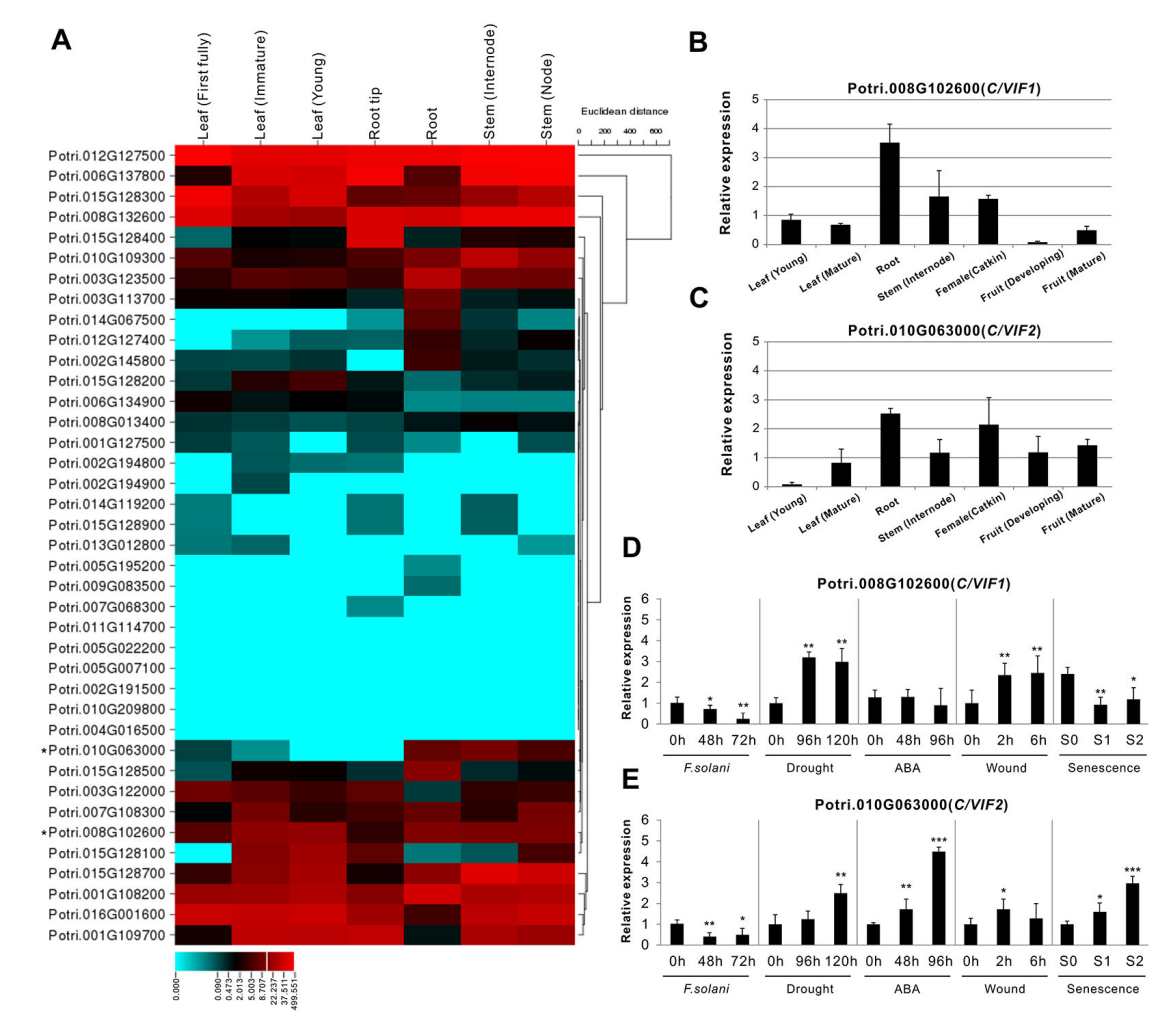
Expression profiles of *PtC/VIF/PMEIs* in various tissues and effects on transcripts of *PtC/VIF1* and *2* upon stress factors. **(A)** Transcriptomic analyses in a heat map showing the transcript abundance of *PtC/VIF/PMEIs* in vegetative tissues of *P. trichocarpa*. **(B**, **C)** qRT-PCR analyses showing the tissue-specific expression and **(D**, **E)** the transcript effects of *PtC/VIF1* and *PtC/VIF2* upon the pathogenic *F. solani*, drought, ABA, wound, and seasonal senescence. The RNA-seq results were given in fragments per kilobase per million reads expression values. The heat map presented for RNA-seq was generated by the online program CIMminer (http://discover.nci.nih.gov/cimminer/home.do). Expression data represent mean values standard error (± SE) of at least three independent biological replicates for qRT-PCR. *PtActin*, *PtUBIC*, and *PtEFα1* were used as reference genes. The asterisks indicate significant differences in comparison with the control using Student’s *t*-test: ***p < 0.001, **p < 0.01, *p < 0.05.

To evaluate the tissue-specific expression patterns, all gene transcripts were examined using RNA-seq and microarray data that were obtained from the Phytozome (v12.1) and eFP database, respectively. The RNA-seq data demonstrated a significant variation of gene expression in vegetative and reproductive tissues. Approximately 12 genes were dominantly expressed in the roots, and more than 10 genes showed high expression levels in the leaves (**Figure 5A**). Interestingly, fourteen genes appeared to be not expressed in the majority of developmental tissues, whereas they showed specific expression in floral tissues (e.g., catkins) (**Supplementary Figure S2**). The gene transcript abundance of all members in tissues were also compared by microarray, which was mostly compatible with the RNA-seq, particularly for those genes with high expression levels in roots (**Supplementary Figure S3**). However, seven C/VIF candidates have not been retrieved their expression patterns owning to the lack of probes for specific targeting (**Supplementary Table S2**). Accordingly, to reinforce the identity of gene expression in various tissues particularly in the roots and leaves, we further conducted the experimental measurement of gene expression levels by qRT-PCR using the *in vitro* cultured plants. A total of 21 *PtC/VIF* candidates were verified their expression levels in the four selected vegetative tissues (roots, stem, young leaves, and mature leaves) (**Supplementary Figure S4**). Among these C/VIF candidates, six of them were identified to be predominantly expressed in the roots and 16 genes were detected the transcript abundance in the mature leaves or young leaves.

### Molecular Characterization of *PtC/VIF1* and *2*

The collectively evolutionary analyses revealed that Potri.008G102600 (PtC/VIF1) and Potri.010G063000 (PtC/VIF2) were identified with significant homologies to the reported orthologous CIFs in tomato and *Arabidopsis*. PtC/VIF1 shows 33.56% protein sequence identities with SlINVINH1 (Jin et al., 2009) and PtC/VIF2 shows 45.75% protein sequence identities with AtCIF1 (Link et al., 2004). PtC/VIF1 and 2 displayed similar genomic patterns and shared 39.04% protein sequence identities. By removing the N-terminal targeting sequences, the deduced mature proteins were comprised of 148 and 146 amino acid residues for PtC/VIF1 and 2, respectively (**Supplementary Table S2**). The predicted MW for the mature PtC/VIF1 is 16.15 kDa with an acid pI of 4.76, and for PtC/VIF2, the MW is 15.69 kDa with a basic pI of 7.02. The multiple sequence alignment revealed that both PtC/VIF1 and 2 contained the motif-1 and the typical hallmarks, four Cys residues. However, only PtC/VIF2 showed the presence of a small motif (PKF) that was defined as a critical sequence for invertase–inhibitor interaction (Hothorn et al., 2010). Accordingly, above RNA-seq and microarray data revealed their extremely high levels of expression in the roots (**Figure 5A** and **Supplementary Figures S2**, **S3**). Further qRT-PCR evaluation of the spatiotemporal expression patterns showed that *PtC/VIF1* was specifically expressed in the roots and stems (**Figure 5B**). By contrast, *PtC/VIF2* displayed predominant expression levels in the roots, followed by in the catkins and fruits (**Figure 5C**). These qRT-PCR results confirmed the tissue-specific expressions through the analyses of RNA-seq and microarray (**Figure 5A** and **Supplementary Figure S2**). The programed stress-related *cis*-regulatory elements within *PtC/VIF1* and *2* promoters allowed us to examine the effects on their expressions upon various environmental factors, including fusarium wilt (*F. solani*), drought, ABA, wound, and senescence. As shown in **Figures 5D and E**, after fungal inoculation of 72 hours, both *PtC/VIF1* and *2* expressions were significantly down-regulated in roots by the pathogenic *F. solani*. Under the drought stress conditions, *PtC/VIF1* showed a constant increase of expressions in the roots, whereas *PtC/VIF2* appeared to be promoted significantly when the time was extended to 96 hours. Additionally, *PtC/VIF2* expression in the roots displayed significant increases under the ABA treatments. By contrast, *PtC/VIF1* expression was markedly induced by the wounding stress. Interestingly, both *PtC/VIF1* and *2* displayed continuous promotions of the expression levels in responses to the seasonal leave senescence.

### Apoplastic Targets of PtC/VIF1 and 2

**Figure 6:**
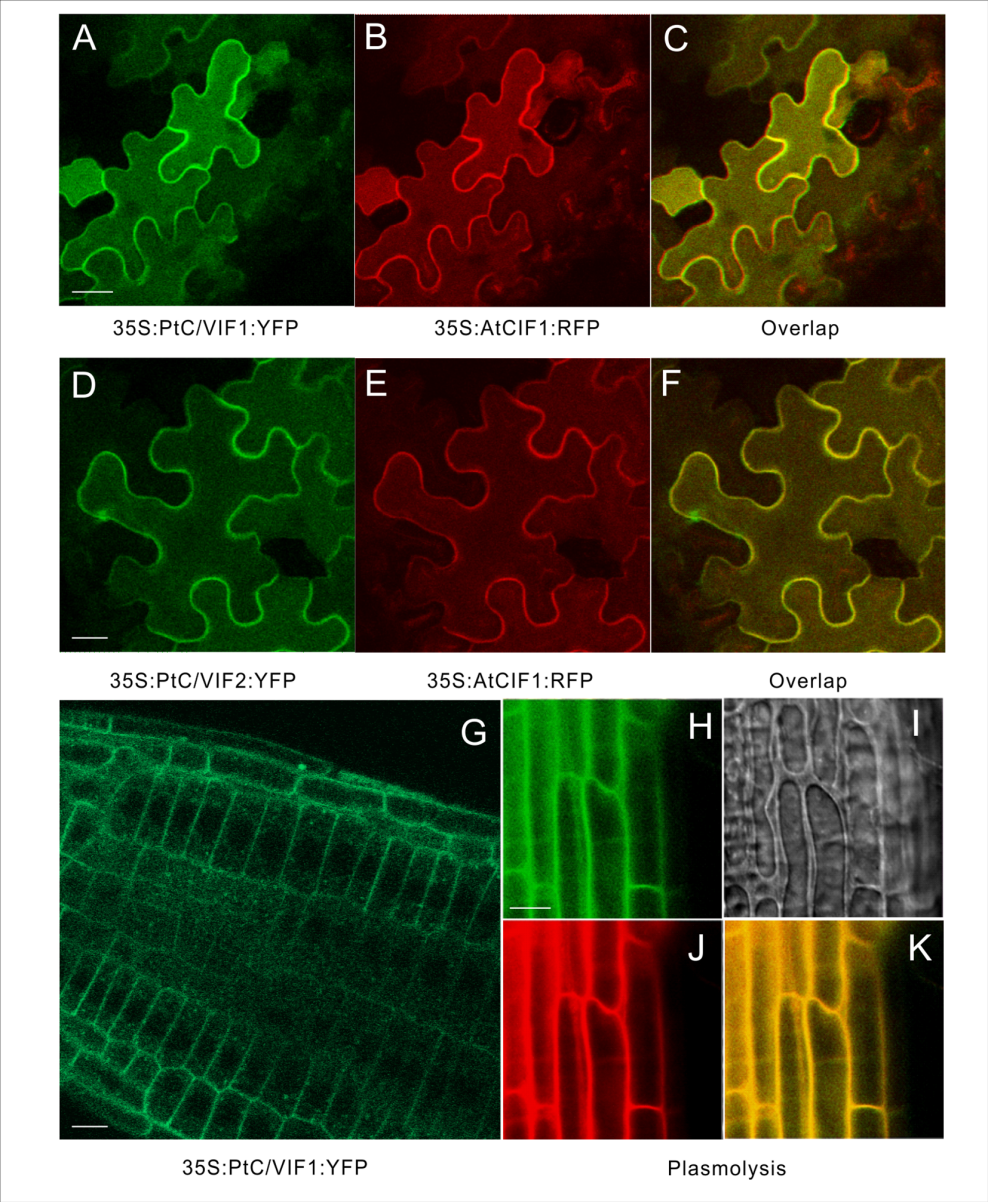
Apoplastic localizations of PtC/VIF1 and 2 in tobacco and *Arabidopsis*. **(A**-**F)** Tobacco leaves were co-infiltrated with *A. tumefaciens* (C58C1) culture harboring the florescent fusion constructs of *35S: PtC/VIF1: YFP* and *35S:AtCIF1: RFP* or *35S: PtC/VIF2: YFP* and *35S:AtCIF1: RFP*. **(A**, **D)** Epidermal cells of tobacco leaf depicting YFP (green) fluorescence. **(B**, **E)** The red fluorescent signals of a cell wall marker AtCIF1. **(C**, **F)** The yellow fluorescent signals captured from the overlap of YFP and RFP fusion. **(G)** Images of CLSM in transgenic *Arabidopsis* showed the yellow fluorescent (green) signal of PtC/VIF1. Fluorescent images showing **(H)** YFP (green) signals, **(I)** the contracted vacuoles, **(J)** PI staining (red), and **(K)** the overlapping signals (yellow) from YFP and PI after plasmolysis (200 mM mannitol). PI (propidium iodide) was used as a marker to track the cell wall for fresh cells. The *Arabidopsis* seedlings grew for five days under short-day conditions and were harvested for the CLSM analysis.

The *in silico* prediction of target sequences of PtC/VIF1 and 2 suggested their subcellular localizations to the apoplast (**Figure 3** and **Supplementary Table S2**). To verify their primary targets, we expressed the fluorescent-labeled proteins in transient and stable transformation systems. For a transient assay, the C-terminal YFP fusion constructs (*35S: PtC/VIF1: YFP* and *35S: PtC/VIF2: YFP*) were co-introduced with a reported *Arabidopsis* cell wall-localization marker (*35S:AtCIF1: RFP*) into tobacco leaf epidermis (**Figures 6A–F**). The overlapped fluorescent signals revealed that distributions of the yellow fluorescence were observed around the cell periphery of the epidermis, suggesting that YFP fusions (green) were fully congruent with that of cells expressing the cell wall marker fused to RFP (red). However, there were no fluorescent signals were visualized from the vacuoles (**Figures 6C, F**). As an alternative approach, the same YFP fusion constructs were stably transformed into *Arabidopsis* and generate transgenic plants. As shown in **Figure 6G**, the YFP signals (green) were captured from the root epidermal cells. After the mannitol-triggered cell plasmolysis, the contracted vacuoles were visualized in the bright field of microscopy (**Figure 6I**). Concurrently, a fluorescent intercalating agent, PI (propidium iodide) was used to stain the cell wall. The captured yellow fluorescent signals from the overlapping of YFP fusion proteins (green) and PI staining (red) suggested that both PtC/VIF1 and 2 were localized to the apoplast (**Figure 6K** and **Supplementary Figure S1E**). Collectively, the image analyses of fluorescent fusions in tobacco leaves and transgenic *Arabidopsis* roots further supported the notion that PtC/VIF1 and 2 primarily targeted to the apoplast.

### Inhibitory Activities of the PtC/VIF1 and 2

**Figure 7:**
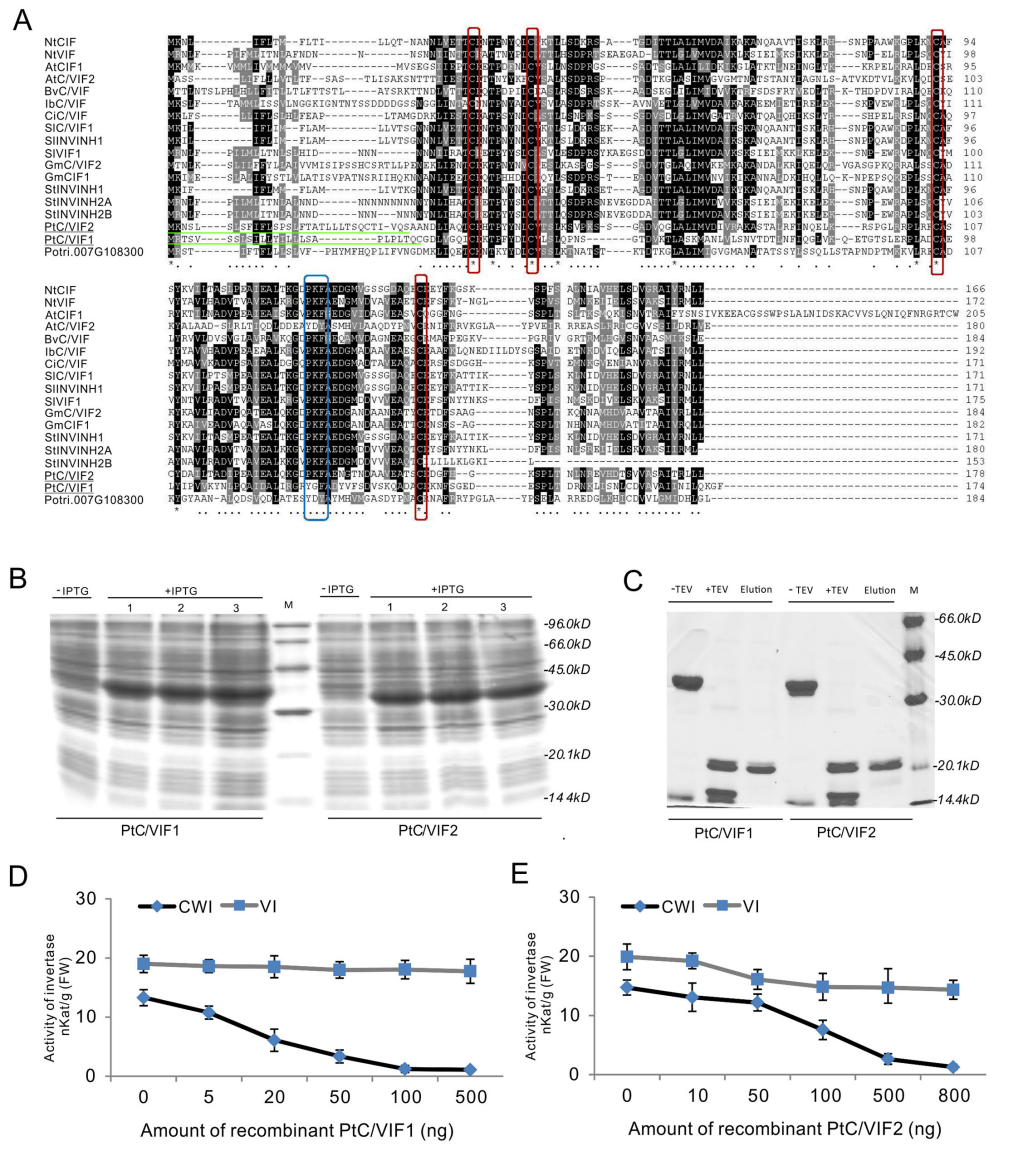
The inhibitory functions *in vitro* of the recombinant PtC/VIF1 and 2. **(A)** Multiple sequence alignment of PtC/VIF homologs showed the presence of targeting sequences (underlined in green), the four Cys residues (boxed in red), and the reported small motif PKF (boxed in blue). **(B**, **C)** SDS-PAGE analyses showed the induction and purification of the recombinant proteins. **(D**, **E)** The functional activities *in vitro* of recombinant proteins were determined by the inhibition of CWI and VI, which were extracted from the roots. The minimum dose input caused maximum inhibitory activities of CWI and VI was 100 ng for the recombinant PtC/VIF1, and 800 ng for the recombinant PtC/VIF2. Determination of the functional enzyme activities represents means ± SE of at least four independent biological replicates.

Given the conserved patterns and apoplastic localization of PtC/VIF1 and 2, we postulated that they might exert the functional inhibition on CWI activities. To specify their enzyme activities and targeting affinities, full-length CDS of *PtC/VIF1* and *2* with the removal of signal peptides were amplified and cloned into the *pETG-20A* vector to generate the 6xHis-tagged N-terminal TrxA-fusion constructs by the heterologous expression in the *E. coli* strain (**Figures 7A, B**). After induction by IPTG (Isopropyl β-d-1-thiogalactopyranoside), the TrxA-fusion proteins were harvested and further released through the cleavage of the TEV protease under the native conditions. As both TrxA and TEV protease contained His-tags, the released recombinant proteins were recovered by Ni-TED affinity chromatography to remove the tagged TrxA and TEV protease. Based on the gel images in **Figure 7C**, the size of finally purified PtC/VIF1 and 2 were close to the deduced MW of mature proteins (**Supplementary Table S2**). Furthermore, under the non-reducing conditions, the observed mobility shifts of recombinant PtC/VIF1 and 2 on SDS-PAGE suggested the synthesis of active intramolecular disulfide bridges (data not shown). To determine the inhibitory targeting activities *in vitro*, different concentrations of recombinant enzymes were incubated with fractions of the root extracted CWI and VI. For PtC/VIF1, the addition of 100 ng its recombinant enzymes demonstrated the maximum inhibition on CWI, showing a significant decrease of 98% activities, whereas no inhibitory effects were detected on VI activities (**Figure 7D**). By contrast, the input of approximately 800 ng of the recombinant PtC/VIF2 led to the maximum inhibition, causing a 95% suppression of CWI activities and, additionally, a 15% decreases of inhibition on VI activities (**Figure 7E**). Both of the recombinant PtC/VIF1 and 2 exhibited remarkably functional affinities on CWI rather than VI, prompting their potential roles as the apoplastic invertase inhibitors *in vitro*.

## Discussion

Emerging reports implicated that CWI and VI exert pivotal roles in maintaining sink capacity and stress acclimation. Since CWI and VI are intrinsically stable enzymes, the regulation of the enzyme activities depends mainly on the post-translational mechanisms that are mediated by the proteinaceous inhibitors (Ruan, 2014). The physiological roles and biotechnology relevance of C/VIF *via* fine-tuning of CWI or VI that modulates sugar metabolism and signaling in apoplast or vacuoles have been attempted in a variety of plants (Greiner et al., 1999; Jin et al., 2009; Liu et al., 2013; Qin et al., 2016; Su et al., 2016; Tang et al., 2017; Chen et al., 2019; Zhao et al., 2019). Hitherto, there is very little literature on molecular mechanisms of the functional genes in the model poplar tree owing to the recalcitrance. The complete genome sequence of *P. trichocarpa* has been released for a decade, and the genetic resource was well-annotated (Tuskan et al., 2006). However, the lack of the molecular basis of a specific gene family has impeded to unveil the physiological significance in the regulation of plant growth and development as well as the potential in the stress adaptation. Thus, the main objective of our work is to extend our knowledge on molecular and biochemical details of C/VIF family in woody plants.

In the present study, to explore the molecular background of C/VIF family genes, we identified a total of 39 candidate genes encoding PtC/VIF in the *Populus* genome (v3.0) of Phytozome 12.1. Analyses of the genomic patterns revealed that they were all intronless genes and had a similar length of the coding region. Approximately 14 gene pairs mapped on 13 chromosomes showed high identities, suggesting that the segmental and tandem duplication occurred commonly during the genome evolution in poplar (**Figures 1A, B**). The genome duplication is an important driver of species origination and diversification, facilitating genes in woody plants to acquire new functions and adapt various environmental factors (Jansson and Douglas, 2007; Bocock et al., 2008). Gene complexity and duplications may give rise to more challenges in the elucidation of functional roles for a unique gene in poplar. In combination with RNA-seq and microarray data, the qRT-PCR analyses of spatiotemporal expression suggested that all candidate genes were differentially expressed in vegetative and reproductive tissues. Additionally, we analyzed the conserved patterns of the PtC/VIF/PMEI protein through the multiple sequence alignment, resulting in the identification of conserved PMEI domain and 15 putative motifs (**Figure 3**). Interestingly, only motif-1 was identified to be evenly distributed among all *PtC/VIF* candidates, whereas other motifs appeared not to spread universally, reflecting that both C/VIF and PMEI family are moderately conserved enzymes.

Based on the phylogenetic comparison between *PtC/VIF* candidates and the functional reported homologs in other plant species, we characterized two putative invertase inhibitors, *PtC/VIF1* and *2*, showing distinguishing features of root-specific expression. The spatiotemporal expressions of CWI coupled with inhibitors and SUTs during the sink organ development have been demonstrated in a variety of plant species, suggesting that the co-expression is a typical pattern underlying the mechanisms of inhibitor-mediated post-translational regulation (Jin et al., 2009; Wang and Ruan, 2012; Su et al., 2016). The co-expression (localization) patterns of invertase and the inhibitor provide the clues for the direct functional target. Such dispersed co-localization also contributes to the efficient transport of the hydrolyzed hexose to the sink fruits *via* modulation of the enzyme activities and sugar signaling (Palmer et al., 2015). Recent reports on evolutionary analyses suggested the presence of five CWI homologs and three VI homologs in the poplar genome (Bocock et al., 2008), which allowed us to reassess their expression patterns in our selected tissues. The RT-PCR validation revealed that three CWI genes (*PtCWI3*, *4*, and *5*) showed transcript abundance in the roots and leaves (**Supplementary Figure S4**). These findings envisioned the potential co-expression of three *PtCWI* genes with *PtC/VIF1* or *2* in poplar normal growth. However, whether the co-expression patterns between inhibitor genes and *CWIs* are critical under the stress regime or which inhibitor(s) would target specific CWI gene(s) *in vivo* remains to be determined further.

As discussed previously, the differential expression profiles of *PtC/VIF1* and *2* in response to various stress factors indicated the complexities and crosstalk of phytohormone and environmental cues (**Figures 5D, E**). Increases in enzyme activities within the apoplastic space upon pathogen infection suggested that CWI served as an essential activator in plant defense regulation (Tauzin and Giardina, 2014). The depression of CIF-encoded gene expression contributed to fortify the hexose capacity, resulting in reduced disease symptoms in apoplastic space (Siemens et al., 2011; Veillet et al., 2016; Su et al., 2018). Under the infection of the *F. solani*, significant down-regulation of *PtC/VIF1* and *2* transcripts were observed after 72 hour inoculation, indicating that they both may be involved in the sucrose-mediated defense pathway. This finding reconciled the ongoing RNA-seq analyses, showing similar patterns of suppressed gene transcript levels among the majorly affected genes in roots with *F. solani* infection (data not shown). Interestingly, some research revealed that the boosting of pathogen innate invertase led to the reprogramed sucrose hydrolysis that may maintain the sugar demand to their benefit (Chang et al., 2017). Collectively, it remains to be deciphered whether CIFs indeed function in a manner of fine-tuning sucrose homeostasis and signaling during plant pathogenesis, or what specific factors and molecular mechanisms potentially perturb the inhibitor gene expression and subsequently, activate/deactivate the immune defense responses to apoplast-adapted stresses (Veillet et al., 2016; Naseem et al., 2017).

The dynamic processes of drought tolerance in plants involved sophisticated control of water influx, cellular osmosis, and sugar metabolism (Golldack et al., 2014). The accumulated storage sugars were also identified to be in correlation with the increase of AI transcripts upon the drought stress (Ji et al., 2010). Under abiotic conditions, the constant induction of *PtC/VIF1* transcript upon drought and wound in the roots suggested that it was dehydration- and wound-responsive gene. Recently, promising work in tomato revealed that significant elevation of CWI activities rather than the transcripts conferred the improvement of drought tolerance (Albacete et al., 2015), reflecting the roles of CIF in the post-translational regulation. Suppression of a tomato *CIF* expression can significantly delay the leaf senescence and fruit size (Jin et al., 2009). An extracellular invertase inhibitor, *AtCIF1* was reevaluated to act as the essential stimulator to be involved in seed germination and biomass control (Su et al., 2016), prompting that the post-translational modulation of CWI positively impacted on sink capacity. In accordance with this, the marked induction of *PtC/VIF2* transcript upon ABA and the seasonal senescence provided clues that *PtC/VIF2* may be a critical component in processing the nitrogen metabolism and remobilization during the leaf aging.

Sugar metabolism is a highly complex network in perennial woody plants and transferred between several intracellular or extracellular compartments for the metabolite biosynthesis, partitioning, and storage (Bocock et al., 2008). In the apoplast, the external supply of carbohydrates is much utilized by sink organs for the developmental and reproductive processes. However, the post-translational mechanisms underlying the regulation of acid invertase through proteinaceous inhibitors have received less attention in woody plants, mostly owing to the lack of extensive molecular basis and the biochemical reports. The recently updated genome assembling in *P. trichocarpa* enables us to mine the C/VIF family for the post-translational modulation in sucrose metabolism and stress response in poplar. Along with the comprehensive view of the genomic patterns and expression profiling, analyses of the phylogenies and conserved motifs revealed that PtC/VIF1 and 2 were closer to the reported C/VIF homologs, suggesting the potential action as invertase inhibitors *in vitro*. Based on the *in silico* analyses, they both were deduced ultimately to transport mature proteins to the apoplast. Thereafter, using the transient and stable expression of YFP-fusion proteins in tobacco and *Arabidopsis*, we observed the fluorescent signals from the cell wall, confirming the typical patterns of apoplast-localized proteins. However, further exploit of their subcellular localization and co-localization with targeting invertase in the cells of native poplar plants remain to be solved.

Accordingly, the comparative crystallographic approach revealed that the target specificity of homologous PMEI and C/VIF used similar structural modules to exert differentially inhibitory functions (Hothorn et al., 2004). However, it is still unreliable to predict the functional pattern from the sequence alone owing to the graded identities of conserved domains and motifs between C/VIF and PMEI family (Link et al., 2004; Zuma et al., 2018). Additional variation of residue combination also may impact on respective interface between C/VIF/PMEI and the targeting proteins (Hothorn et al., 2010), prompting the situation that the use of direct enzyme assay may be the optimized way to distinguish C/VIF from PMEI prior to unveiling the physiological roles in the regulation of plant development and stress tolerance (Wolf et al., 2003; Link et al., 2004). Thus, to examine the specific enzyme properties and targeting affinities, a functional inhibition assay was implemented through the heterologous expression and purification of recombinant proteins in *E.coli*. Based on the functional determination of the enzyme activities, PtC/VIF1 and 2 were confirmed to exhibit a large proportion of inhibitory activities on the extracted CWI rather than VI, further corroborating their roles as the genuine apoplastic invertase inhibitors.

## Conclusions

Thus far, there has been ongoing interest in the improvement of poplar performance with strengthened pathogen resistance and stress tolerance remains a significant challenge for modern agriculture and forestry. Accumulated evidence has prompted that the small inhibitory proteins exert fundamental roles in plant growth and development as well as the regulation of sucrose metabolism and homeostasis through the fine-tuning of the acid invertase activities. Here, we described molecular and genetic details of PtC/VIF family is essential to implicate how genes influence the phenotypes. The spatiotemporal expression patterns of PtC/VIF-encoded genes may confer functional specificity and diversity in response to stress stimuli and environmental cues in woody plants. Among these candidates, *PtC/VIF1* and *2* represent the first invertase inhibitor genes to be characterized in woody plants. Taken together, a remarkable feature of functional PtC/VIF1 and 2 contribute to in-depth unraveling the roles *in vivo* and the post-translational mechanisms underlying the molecular interaction with their targeting enzymes. Further work will attempt to evaluate the possible phenotypes of genetically constructed mutants under stress exposure, and in the long term, it may facilitate the increases of apoplast-adapted pathogen infection and diverse abiotic stressors.

## Data Availability Statement

The microarray data: GEO: GSE13990 (https://www.ncbi.nlm.nih.gov/gds/?term=GSE13990); BioProject: PRJNA112485 (https://www.ncbi.nlm.nih.gov/bioproject/?term=GSE13990. 15.01.2009). The RNA-seq data: Accession number: AARH00000000.3 (30.11.2018); BioProject: PRJNA10772 (https://www.ncbi.nlm.nih.gov/bioproject/PRJNA10772/), GCF_000002775.4 (https://www.ncbi.nlm.nih.gov/assembly/GCA_000002775.3 24.01.2018) contains a large number of RNA-seq data, of which, the accession number corresponding to our manuscript in SRA is SRP077540 (https://www.ncbi.nlm.nih.gov/sra/?term=SRP077540), including the detailed different SRA_run (Leaf_FFE: SRR3727130, 32, and 40; Leaf_Immature: SRR3727123, 25, and 39; Leaf_Young: SRR3727116, 21, and 33; Stem_Node: SRR3727110, 17, and 38; Stem_Inode: SRR3727120, 24, and 41; Roots: SRR3727119, 35, and 36; Roottip: SRR3727111, 15, and 22.

## Author Contributions

TS and MH designed the experiment, collected and analyzed all of the data. TS and MH prepared the initial draft of the manuscript and developed the concept. MH and YF were responsible for approving the final draft of the manuscript. TS conducted the image analyses of CLSM and protein purification. JM and HZ performed the qRT-PCR analysis and enzyme assay. QZ and JZ assisted JM and HZ with the experiment conduction. HZ was responsible for plant culture *in vitro*. TS did much work on the bioinformatics analysis, including conserved domain and promoter analyses, RNA-seq collection, and heat map construction. All authors have reviewed the manuscript.

## Funding

This research was supported by the Natural Science Foundation of China (NSFC) (31870589; 31700525), the Natural Science Foundation of Jiangsu Province (NSFJ) (BK20170921), the Scientific Research Foundation for High-Level Talents of Nanjing Forestry University (SRFNFU) (GXL2017011; GXL2017012), the Priority Academic Program Development of Jiangsu Higher Education Institutions (PAPD), and the Undergraduate Innovation and Entrepreneurship Training Programs in NFU (2018NFUSPITP044).

## Conflict of Interest

The authors declare that the research was conducted in the absence of any commercial or financial relationships that could be construed as a potential conflict of interest.
