# Supplementary material for: Functional Characterization of Invertase Inhibitors PtC/VIF1 and 2 Revealed Their Involvements in the Defense Response to Fungal Pathogen in Populus trichocarpa
Source: Front Plant Sci. 2020 Jan 8;10:1654. doi: 10.3389/fpls.2019.01654 (PMC6960229; doi:10.3389/fpls.2019.01654)
Supplement: Supplementary file 1 [file DataSheet_1.docx]

**TABLE S1.** **List of primers used for analyses of qRT-PCR, subcellular targeting, and recombinant protein expression.**

| **Primer Name** | **Sequence** | | | | **Access number/Gene name** | | |
| --- | --- | --- | --- | --- | --- | --- | --- |
| **qRT-PCR** | | | | | | | |
| *β-Actin_qF* | | GACCTTCAATGTGCCTGCAA | | | | Potri.019G006700.1/*Ptβ-Actin* | |
| *β-Actin_qR* | | ACCATCACCAGAATCCAGCA | | | |  |  |
| *EF1𝛼_qF* | | TCCGTCTTCCACTTCAGGATGTCT | | | | Potri.006G130900.1/*PtEF1𝛼* | |
| *EF1𝛼_qR* | | GTCACGACCATACCAGGCTTCAG | | | |  |  |
| *UBIC_qF2* | | TTGCTTGAGGAACTTGAAC | | | | Potri.006G205700.7/*PtUBIC* | |
| *UBIC_qR2* | | GTGAGGACCGATTACAGT | | | |  |  |
| *qF-51* | | TCACCAACAACACAGTGCTTC | | | | Potri.001G108200.1 | |
| *qR-51* | | AGTCACGTCACAGACCGTTT | | | |  |  |
| *qF-2* | | ATTGTTGCGCCTGTTACTGC | | | | Potri.001G109700.1 | |
| *qR-2* | | AAGAAGGGACTGCCAAGGAC | | | |  |  |
| *qF-3* | | CGTACTTCGGTGAAAGCAGC | | | | Potri.001G127500.1 | |
| *qR-3* | | TTGGCGGAAGATTGCGTTTC | | | |  |  |
| *qF-4* | | GGGCCTATATCGAGACTGCG | | | | Potri.002G145800.1 | |
| *qR-4* | | TCACGGATAAGGCAGCTTGG | | | |  |  |
| *qF-9* | | ACACGTCGGAGGTGCATTAG | | | | Potri.003G122000.1 | |
| *qR-9* | | CGTAGAATCATTTGCAGCACTGT | | | |  |  |
| *qF-10* | | AGCCCTCACCAACAGGAAAG | | | | Potri.003G123500.1 | |
| *qR-10* | | CTGGTGAAGCAGGAGGATGG | | | |  |  |
| *qF-15* | | TTGCAAATCGCCGGAAAAGA | | | | Potri.006G134900.1 | |
| *qR-15* | | TCTGATGTTTGGCTCCGAGG | | | |  |  |
| *qF-17* | | GCCACGGCCACATCATCTTA | | | | Potri.007G108300.1 | |
| *qR-17* | | TGGATAATCCGAGGCTCCCA | | | |  |  |
| *qF-19* | | GTACCCCAGATGCCGATAGC | | | | Potri.008G013400.1 | |
| *qR-19* | | TACCTGCCAACTCAGCCAAC | | | |  |  |
| *qF-48* | | TAGAGAGGCCACTGGCTGAT | | | | Potri.008G102600.1/*PtC/VIF2* | |
| *qR-48* | | ATGGTGATTCATCCTCGCCC | | | |  |  |
| *qF-21* | | CGTATCCAACCTAACCCGCC | | | | Potri.008G132600.1 | |
| *qR-21* | | TTTCCGCATCTGCTTCAACG | | | |  |  |
| *qF-23* | | TCCCGGAAGCTATTGAAGCC | | | | Potri.010G063000.1/*PtC/VIF1* | |
| *qR-23* | | GAGATTTGCCGTGGAAACCG | | | |  |  |
| *qF-28* | | CTGCCTTGTGTGTGCAATCC | | | | Potri.012G127500.1 | |
| *qR-28* | | ACAAGCCTGTCCACGGTATC | | | |  |  |
| *qF-36-1* | | CATCCTCGGCAGCTATCACC | | | | Potri.015G128500.1 | |
| *qR-36-1* | | GCTAGAAGCTTTGAGGGCCA | | | |  |  |
| *qF-54* | | GACGTCTAGCTCTCACTGCC | | | | Potri.003G113700.1 | |
| *qR-54* | | CAACAGCTCGACCCGTATGA | | | |  |  |
| *qF-58* | | GGCAGCCTAAAAGGTTCCGA | | | | Potri.012G127400.1 | |
| *qR-58* | | GTCAGCCTGGCAACATTCAC | | | |  |  |
| *qF-59* | | TGTGGCTTGACCGATTACCC | | | | Potri.014G119200.1 | |
| *qR-59* | | GTTTGCCAATGTCGTGGGAG | | | |  |  |
| *qF-63* | | GCCTCAGGTTGAGTCTGGTG | | | | Potri.015G128400.1 | |
| *qR-63* | | CGACGCATTCCTTTATGGCG | | | |  |  |
| *qF-64* | | GCCTAGACAAGGCTCAGTCC | | | | Potri.015G128700.1 | |
| *qR-64* | | AATCCTGGCCCTTGGATGAC | | | |  |  |
| *qF-66* | | TGGCTATGCCACACACTCTC | | | | Potri.016G077400.1/*PtCWI1* | |
| *qR-66* | | GTAGTACATAGGCGCTGCGT | | | |  |  |
| *qF-67* | | TAGGAACTGGATCAACGGCG | | | | Potri.016G077500.1/*PtCWI2* | |
| *qR-67* | | GACAAGTTTGCGGGTACTGC | | | |  |  |
| *qF-42* | | AGGAGCTCTTCCTTGGGAGT | | | | Potri.006G210600.1/*PtCWI3* | |
| *qR-42* | | CTCTACAGTGACGGTCTCGC | | | |  |  |
| *qF-68* | | TAGTGCAGTGGCCCATTGAG | | | | Potri.006G227500.1/*PtCWI4* | |
| *qR-68* | | AACCTCCACATCTGCCTGTG | | | |  |  |
| *qF-44* | | TGGTGTGGATCTGCGGTATG | | | | Potri.006G227400.1/*PtCWI5* | |
| *qR-44* | | GACTGAAGTCCAGACCAGCC | | | |  |  |
| *qF-45* | | GACGGTGCCACACAACTAGA | | | | Potri.003G126300.1/*PtVI1* | |
| *qR-45* | | ACCAGAAGACCAAACGGTCC | | | |  |  |
| *qF-69* | | CCCATCTCCGACGATCCAAG | | | | Potri.003G112600.1/*PtVI2* | |
| *qR-69* | | TCACGTTGGCCTTCTCTGAC | | | |  |  |
| *qF-70* | | GTTCGGTTGTGCCACTTGAC | | | | Potri.015G127100.1/*PtVI3* | |
| *qR-70* | | ATGGTCCCAAAGCACTCCTG | | | |  |  |
| **Subcellular (co-)localization** | | | | | | | |
| *qF-49-attB1* | | | GGGGACAAGTTTGTACAAAAAAGCAGGCTCCATGAGAACTTCAGTCTCTTC | | | | Potri.008G102600.1  *PtC/VIF2-pB7YWG2.0* |
| *qR-49-attB2stop(-)* | | | GGGGACCACTTTGTACAAGAAAGCTGGGTC  GAAACCCTTCTGCAAT | | | |  |
| *qF-50-attB1* | | | GGGGACAAGTTTGTACAAAAAAGCAGGCTCCATGAAGAATTCTCTGTCC | | | | Potri.010G063000.1  *PtC/VIF1-pB7YWG2.0* |
| *qR-50 attB2stop(-)* | | | GGGGACCACTTTGTACAAGAAAGCTGGGTC  TCAAAGTAACAGCCTGGTGA | | | |  |
| *AtCIF1_attB1* | | | GGGGACAAGTTTGTACAAAAAAGCAGGCTCC  ATGAAGATGATGAAGGTGATGATG | | | | At1g47960  *AtCIF1- pK7RWG2.0* |
| *AtCIF1_attB2stop(-)* | | | GGGGACCACTTTGTACAAGAAAGCTGGGTC  GAGCACCACCAACAAGTTCTTCCTCTATTGAA  ACAAGT  TCTTCCTCTATTGAA  CCAACAAGTTCTTCCTCTATTGAA | | | |  |
| *pDONR201F* | | | TCGCGTTAACGCTAGCATGGATCTC | | | |  |
| *pDONR201R* | | | GTAACATCAGAGATTTTGAGACAC | | | |  |
| **Protein expression in *E.coli*** | | | | | | | |
| *attB1-adaptor-Tev* | | | | GGGGACAAGTTTGTACAAAAAAGCAGGCT CTGAGAATCTTTATTTTCAGGGC | | |  |
| *attB2-adaptor* | | | | GGGGACCACTTTGTACAAGAAAGCTGGGT | | |  |
| *PtC/VIF2-F(sp-)*  AttB1_TEVFor | | | | TATTTTCAGGGCTCCGGTGATCTAGTAGGCC | | | *pETG20A-PtC/VIF2* |
| *PtC/VIF2-R* | | | | AGAAAGCTGGGTTTCAGAAACCCTTCTGC | | |  |
| *PtC/VIF1-F(sp-)*  AttB1_TEVFor | | | | TATTTTCAGGGCTCCGCTGCCAATGATCTGATAG | | | *pETG20A-PtC/VIF1* |
| *PtC/VIF1-R* | | | | AGAAAGCTGGGTTTCATCAAAGTAACAG | | |  |

**TABLE S2. Putative C/VIF candidate genes in *P. trichocarpa.***

| **Gene ID**  **(v3.0)** | **NCBI**  **Accession N** | **Probe for Microarray** | **Chr location**  **(5'-3')** | **gDNA**  **(bp)** | **ORF**  **(bp)** | **Trps** | **Size (A.A)** | **MW (kDa)** | **PI** | **SP** | **Target** |
| --- | --- | --- | --- | --- | --- | --- | --- | --- | --- | --- | --- |
| Potri.001G108200 | XM_002298018.3 | PtpAffx.84646.1.S1_at | Chr1:8591512-8592765 | 1254 | 723 | 1 | 240 | 26.49 | 5.08 | 1-53 | Apo |
| Potri.001G109700 | XM_002298025.3 | Ptp.5286.1.S1_at | Chr1:8716781-8717831 | 1051 | 558 | 1 | 185 | 20.54 | 5.45 | 1-22 | Apo |
| Potri.001G127500 | XM_006368186.2 | PtpAffx.220129.1.S1_at | Chr1:10335804-10336343 | 540 | 540 | 1 | 179 | 19.32 | 5.00 | 1-27 | Apo |
| Potri.002G145800 | XM_002302491.3 | PtpAffx.107621.1.A1_at | Chr2:10868644-10869440 | 797 | 615 | 1 | 204 | 22.56 | 9.32 | 1-24 | Apo |
| Potri.002G191500 | XM_002301496.3 | PtpAffx.202400.1.S1_at | Chr2:15181110-15181670 | 561 | 561 | 1 | 186 | 20.30 | 5.65 | 1-27 | Apo |
| Potri.002G194800 | XM_006386652.2 | PtpAffx.202382.1.S1_s_at | Chr2:15578045-15578761 | 717 | 717 | 1 | 238 | 24.72 | 4.63 | 1-21 | Apo |
| Potri.002G194900 | XM_006388381.2 | PtpAffx.202382.1.S1_s_at | Chr2:15600246-15600962 | 717 | 717 | 1 | 238 | 24.78 | 4.81 | 1-21 | Apo |
| Potri.003G113700 | XM_024597148.1 | Ptp.8039.1.S1_at | Chr3:13733135-13734152 | 1018 | 597 | 1 | 198 | 20.76 | 10.14 | 1-24 | Apo |
| Potri.003G122000 | XM_002304435.3 | PtpAffx.69725.1.S1_a_at | Chr3:14429594-14430325 | 732 | 549 | 1 | 182 | 20.34 | 5.79 | 1-20 | Apo |
| Potri.003G123500 | XM_002304441.3 | PtpAffx.84646.1.S1_at | Chr3:14521527-14522506 | 980 | 723 | 1 | 240 | 26.60 | 4.47 | 1-53 | Apo |
| Potri.004G016500 | XM_002304878.2 | PtpAffx.203681.1.S1_at | Chr4:1114524-1115087 | 564 | 564 | 1 | 187 | 20.24 | 7.61 | 1-25 | Apo |
| Potri.005G007100 | XM_006386652.2 | PtpAffx.215887.1.S1_at | Chr5:413394-413939 | 546 | 546 | 1 | 181 | 20.30 | 5.42 | 1-27 | Apo |
| Potri.005G022200 | n.a | PtpAffx.214988.1.S1_s_at | Chr5:1711706-1712224 | 519 | 519 | 1 | 172 | 18.47 | 8.90 | 1-26 | Apo |
| Potri.005G195200 | n.a | PtpAffx.205620.1.S1_at | Chr5:21201097-21201657 | 561 | 561 | 1 | 186 | 20.29 | 9.30 | 1-27 | Apo |
| Potri.006G134900 | XM_006381483.2 | PtpAffx.219724.1.S1_at | Chr6:11129451-11130011 | 561 | 561 | 1 | 186 | 20.28 | 4.91 | 1-26 | Apo |
| Potri.006G137800 | XM_006381512.2 | Ptp.6699.1.S1_s_at | Chr6:11466239-11467284 | 1046 | 615 | 1 | 204 | 22.82 | 9.30 | 1-31 | Apo |
| Potri.007G108300 | XM_024605295.1 | PtpAffx.5307.3.A1_a_at | Chr7:13183567-13184482 | 916 | 555 | 1 | 184 | 20.46 | 6.94 | 1-21 | Apo |
| Potri.007G068300 | XM_002310052.2 | PtpAffx.207214.1.S1_at | Chr7:8957188-8957814 | 627 | 543 | 1 | 180 | 20.00 | 9.14 | 1-23 | Apo |
| Potri.008G013400 | XM_006379377.2 | PtpAffx.95403.1.A1_at | Chr8:728027-728948 | 922 | 555 | 1 | 184 | 20.39 | 5.12 | 1-30 | Apo |
| Potri.008G102600 | XM_002311341.3 | PtpAffx.32992.1.S1_at | Chr8:6514558-6515618 | 1061 | 525 | 1 | 174 | 18.97 | 4.92 | 1-26 | Apo |
| Potri.008G132600 | XM_002312415.3 | PtpAffx.58139.1.S1_at | Chr8:8771094-8772054 | 961 | 639 | 1 | 212 | 22.68 | 5.60 | 1-23 | Apo |
| Potri.009G083500 | XM_002313897.2 | PtpAffx.204758.1.S1_at | Chr9:7860767-7861551 | 785 | 528 | 1 | 175 | 19.76 | 9.18 | 1-21 | Apo |
| Potri.010G063000 | XM_002314485.3 | Ptp.3748.1.S1_at | Chr10:9103329-9104033 | 705 | 537 | 1 | 178 | 19.16 | 7.60 | 1-32 | Apo |
| Potri.010G109300 | XM_002314759.3 | n.a | Chr10:12861817-12862691 | 875 | 639 | 1 | 212 | 22.68 | 5.12 | 1-25 | Apo |
| Potri.010G209800 | XM_002315208.2 | PtpAffx.209350.1.S1_at | Chr10:19905536-19906066 | 531 | 531 | 1 | 176 | 19.14 | 5.12 | 1-25 | Apo |
| Potri.011G114700 | XM_002316840.3 | PtpAffx.209947.1.S1_at | Chr11:13937649-1393822 | 573 | 573 | 1 | 190 | 21.06 | 6.27 | 1-22 | Apo |
| Potri.012G127400 | XM_002318844.3 | PtpAffx.30927.1.A1_at | Chr12:14511829-14512641 | 813 | 609 | 1 | 202 | 21.90 | 8.47 | 1-35 | Apo |
| Potri.012G127500 | XM_002318247.3 | Ptp.6758.1.A1_at | Chr12:14516562-14517551 | 990 | 597 | 1 | 198 | 21.42 | 9.60 | 1-22 | Apo |
| Potri.013G012800 | XM_024583813.1 | n.a | Chr13:840867-841603 | 831 | 519 | 1 | 172 | 18.42 | 5.14 | 1-27 | Apo |
| Potri.014G067500 | XM_002320680.3 | PtpAffx.21166.1.S1_at | Chr14:5496455-5497331 | 877 | 618 | 1 | 205 | 22.90 | 9.68 | 1-21 | Apo |
| Potri.014G119200 | XM_002320253.3 | PtpAffx.211712.1.S1_at | Chr14:9249255-9249980 | 726 | 726 | 1 | 241 | 25.39 | 5.02 | 1-23 | Apo |
| Potri.015G128100 | XM_002322367.3 | n.a | Chr15:14006920-14007612 | 693 | 612 | 1 | 203 | 21.69 | 5.89 | 1-28 | Apo |
| Potri.015G128200 | XM_002322368.3 | n.a | Chr15:14013198-14014103 | 2405 | 588 | 1 | 195 | 21.14 | 6.41 | 1-25 | Apo |
| Potri.015G128300 | XM_002322369.3 | Ptp.5816.1.S1_at | Chr15:14020411-14021514 | 2604 | 594 | 1 | 197 | 21.23 | 7.70 | 1-25 | Apo |
| Potri.015G128400 | XM_002321799.3 | Ptp.857.1.S1_at | Chr15:14028406-14029409 | 2504 | 612 | 1 | 203 | 22.43 | 4.64 | 1-33 | Apo |
| Potri.015G128500 | XM_002321800.3 | n.a | Chr15:14034481-14035255 | 775 | 627 | 1 | 208 | 22.50 | 9.14 | 1-35 | Apo |
| Potri.015G128700 | XM_002322370.3 | Ptp.3327.1.S1_at | Chr15:14042637-14043724 | 1088 | 600 | 1 | 199 | 21.82 | 9.50 | 1-34 | Apo |
| Potri.015G128900 | XM_024586017.1 | PtpAffx.213024.1.S1_at | Chr15:14054001-14054622 | 622 | 600 | 1 | 199 | 21.62 | 8.53 | 1-33 | Apo |
| Potri.016G001600 | XM_006373493.2 | Ptp.4540.2.S1_s_at | Chr16:76264-77844 | 3081 | 561 | 1 | 186 | 20.34 | 8.53 | 1-30 | Apo |

The gene ID was obtained from the genome assembly nomenclature of in JGI gene catalog in Phytozome v12.1 (https://phytozome.jgi.doe.gov/pz/portal.html) and GenBank (https://www.ncbi.nlm.nih.gov/). Chr, chromosome; ORF, open reading frame; Trps, transcripts; AA, amino acid; MW, deduced molecular weight (kDa); pI: isoelectric point; CDS, coding DNA sequence; bp, base pair; n.a, not applicable. Apo, Apoplast; Prediction of protein subcellular targeting was performed by programs of PSORT (https://wolfpsort.hgc.jp/) and Phobius (http://phobius.binf.ku.dk/). Protein molecular weight (MW) and isoelectric point (pI) were deduced by the program of ExPASy (http://web.expasy.org/compute_pi/).


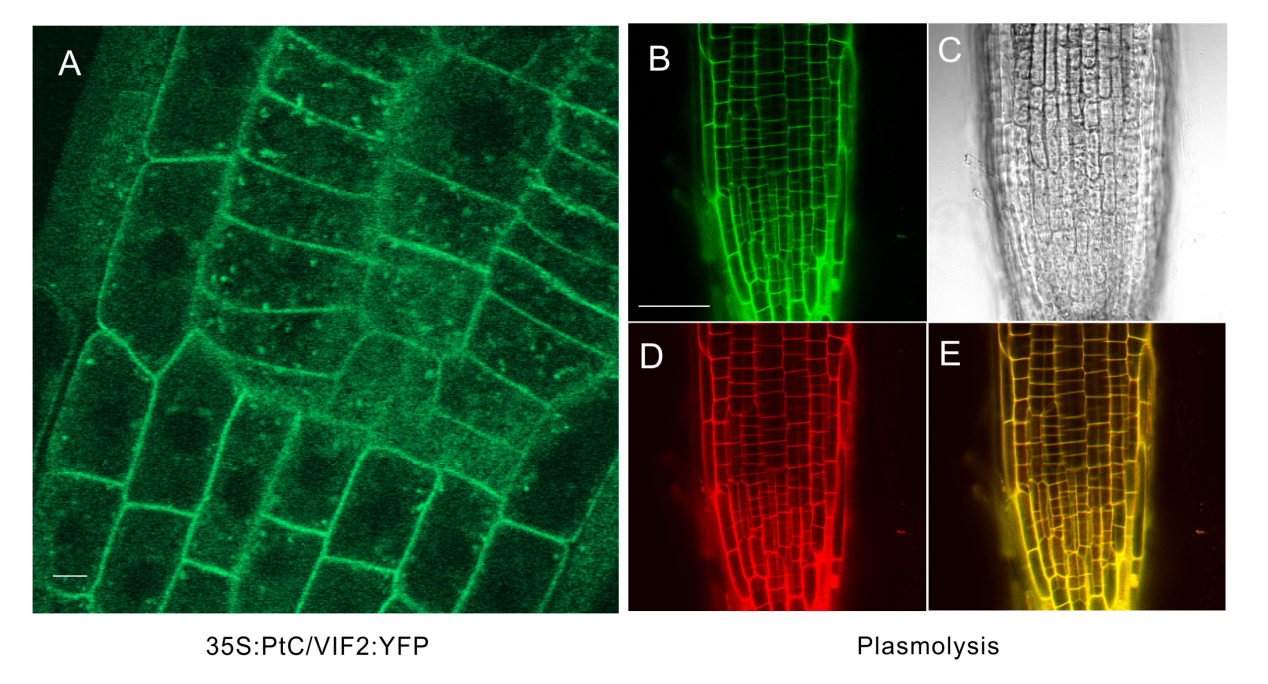


**FIGURE S1. Apoplastic localization of PtC/VIF2 in transgenic *Arabidopsis* roots.** (**A**) CLSM image of *35S:PtC/VIF2:YFP*. Images of CLSM in transgenic *Arabidopsis* roots showed the apoplastic localization of PtC/VIF2 (**B**) and co-localization with PI (propidium iodide) staining after the plasmolysis with mannitol (**C-E**). *Arabidopsis* seedlings grew for five days under short-day conditions and were harvested for image analysis.


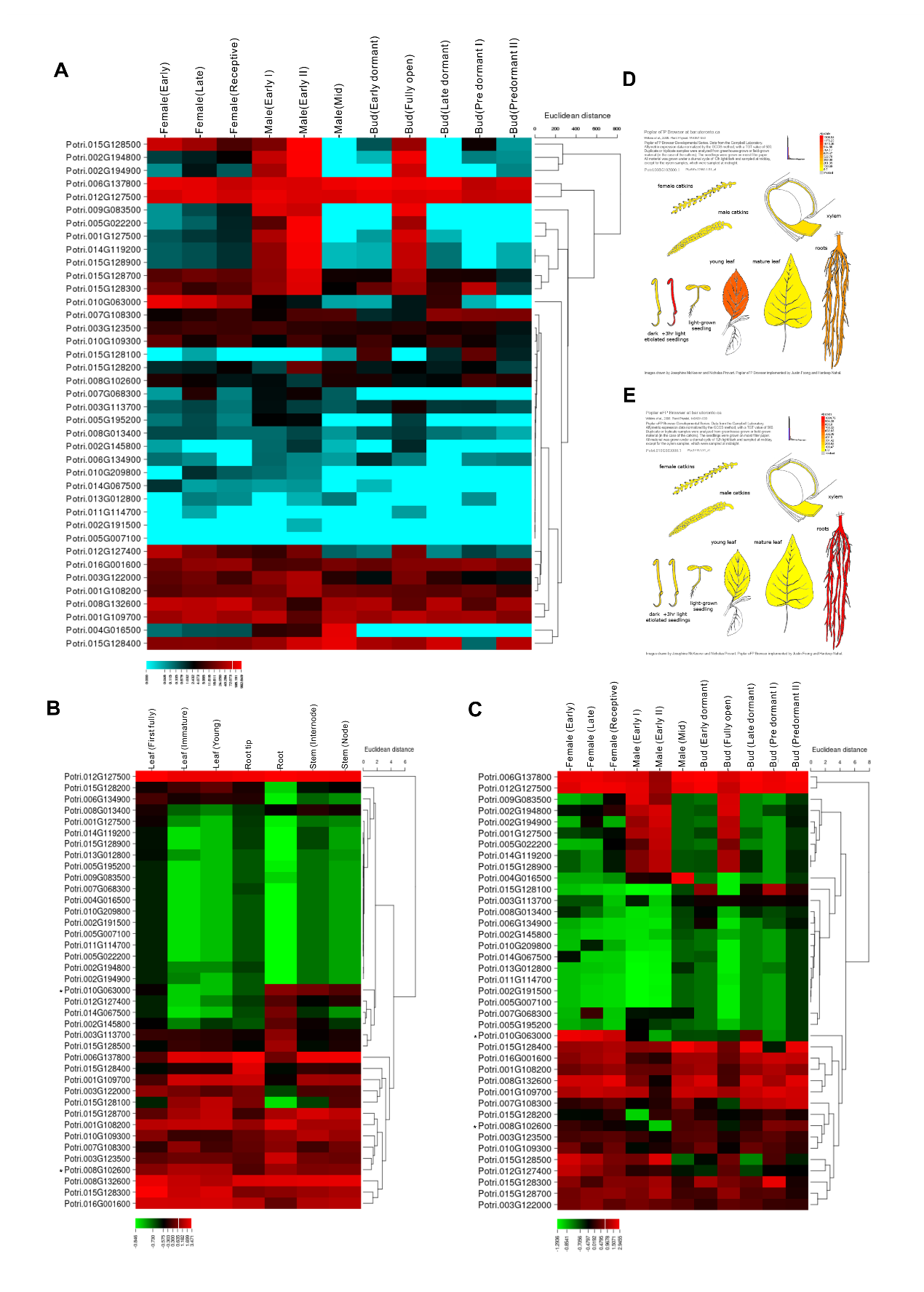


**FIGURE S2**. **Expression profiles of *PtC/VIF/PMEI* genes in various tissues of *P. trichocarpa***. (**A**) Transcriptomic analyses in a heat map showing the transcript abundance in reproductive tissues. The RNA-seq results were given in FPKM (fragments per kilobase per million reads) expression values. The heat map showing the relative transcript levels of genes in vegetative tissues (**B**) and reproductive tissues (**C**) after the value was processed by Z-score calculation. Images show the transcript abundance of *PtC/VIF1* (**D)** and *PtC/VIF2* **(E**) in the roots by microarray analyses.

**
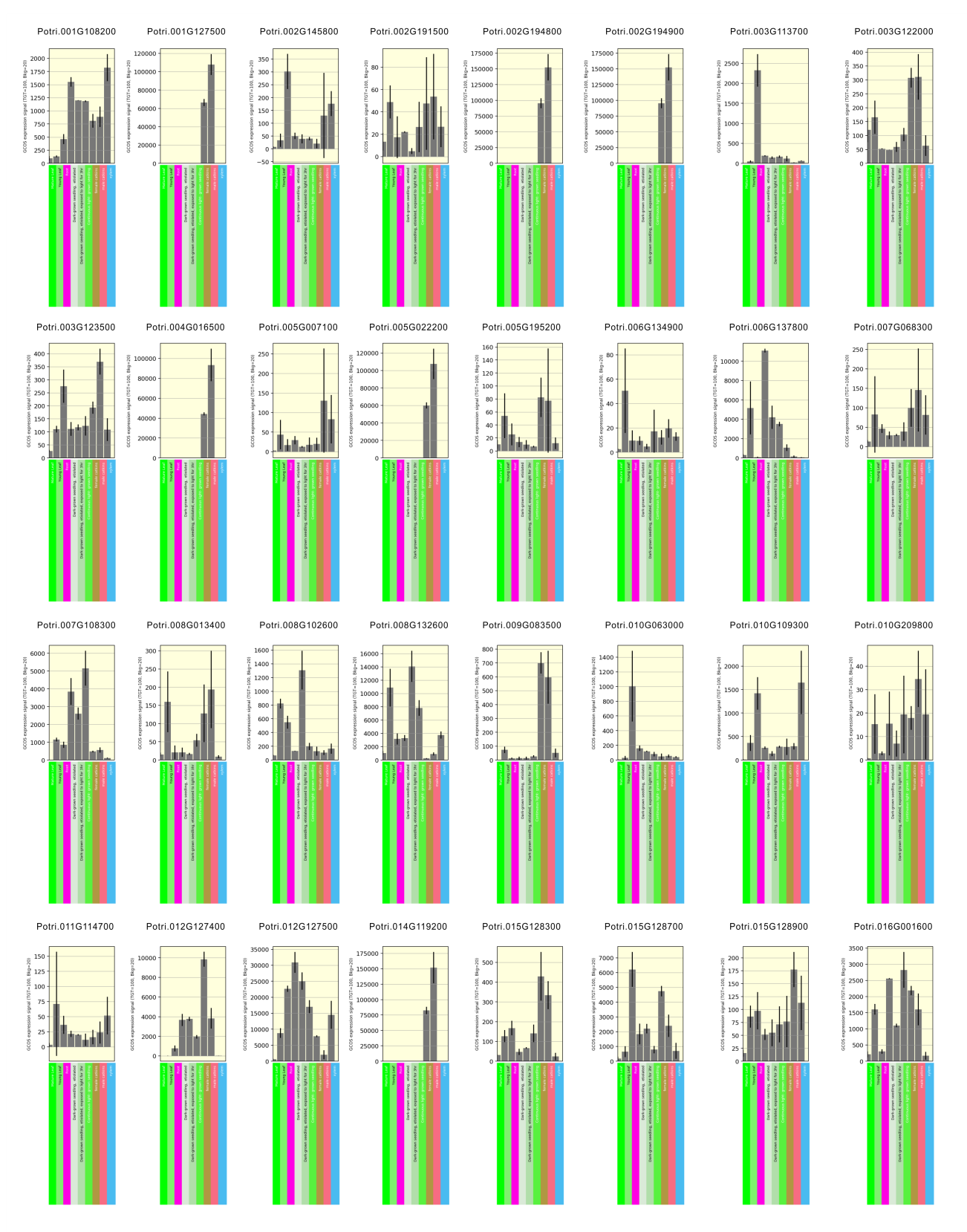
**

**FIGURE S3**. **Microarray analyses of *PtC/VIF/PMEIs* in various tissues of *P. trichocarpa*.** The Affymetrix expression data was accessible from the database of Poplar eFP Browser (http://bar.utoronto.ca/efppop/cgi-bin/efpWeb.cgi). Duplicate or triplicate samples of *P. trichocarpa* were used for the microarray analysis.


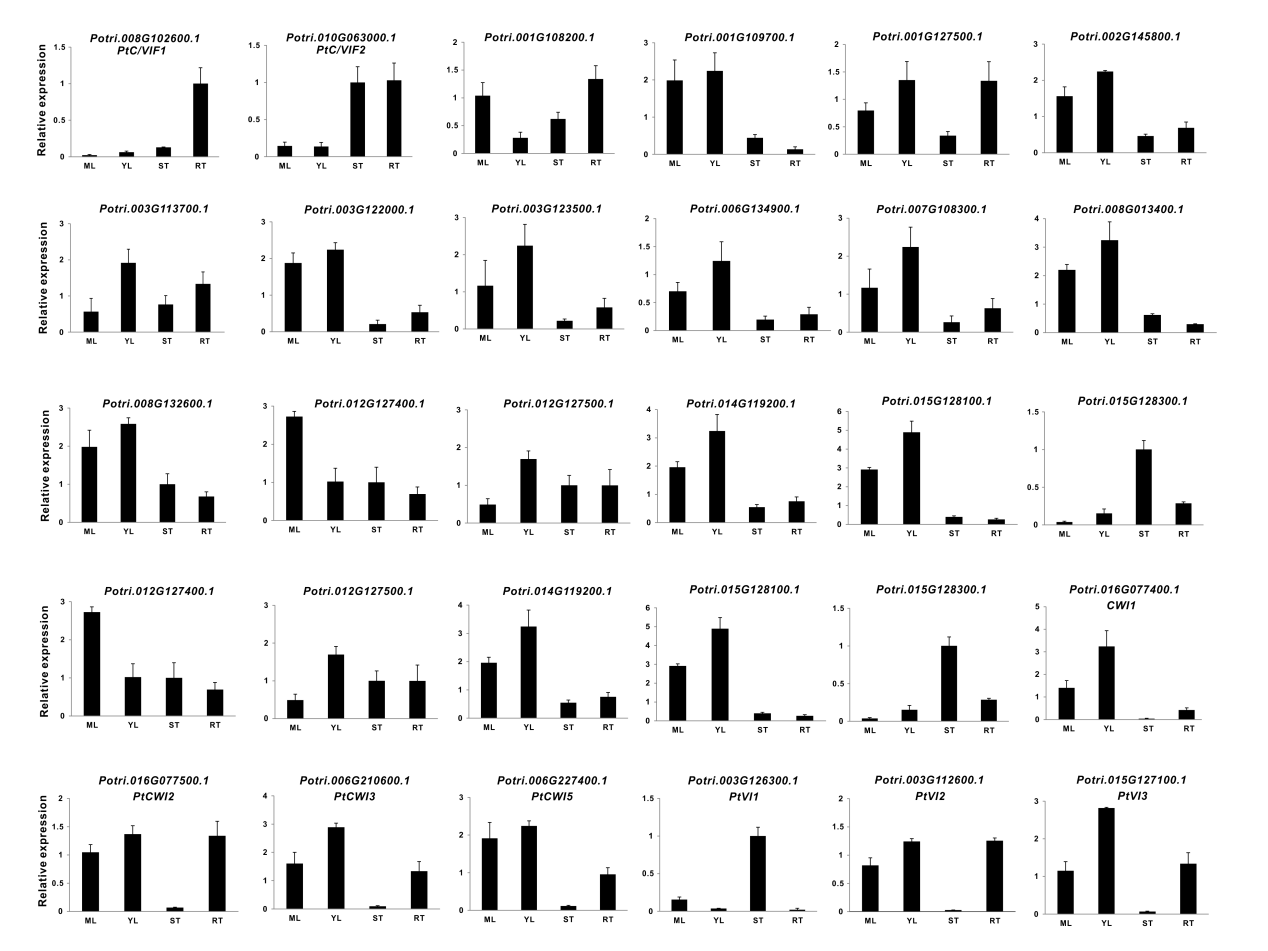


**FIGURE S4**. **Tissue-specific expression of *PtC/VIF/PMEIs* in tissue cultured plants**. Samples were harvested from the ML (mature leaf), YL (young leaf), ST (stem), and RT (root). Data represent mean values standard error (±SE) of at least three independent biological replicates for qRT-PCR. *PtActin*, *PtUBIC*, and *PtEFα1* were used as reference genes.
